# Marine medaka ATP-binding cassette (ABC) superfamily and new insight into teleost Abch nomenclature

**DOI:** 10.1038/srep15409

**Published:** 2015-10-16

**Authors:** Chang-Bum Jeong, Bo-Mi Kim, Hye-Min Kang, Ik-Young Choi, Jae-Sung Rhee, Jae-Seong Lee

**Affiliations:** 1 Department of Biological Science, College of Science, Sungkyunkwan University, Suwon 16419, South Korea; 2 Department of Chemistry, College of Natural Sciences, Hanyang University, Seoul 04763, South Korea; 3 National Instrumentation Center for Environmental Management, College of Agriculture and Life Sciences, Seoul National University, Seoul 08826, South Korea; 4 Department of Marine Science, College of Natural Sciences, Incheon National University, Incheon 22012, South Korea

## Abstract

The ABC gene family is recognized as one of the largest gene families in all kingdoms of life. Although many genes involved in the ABC superfamily have been annotated from several fish species, information on large sets of the ABC superfamily and their evolutionary characterization are still unclear. In the marine medaka *Oryzias melastigma*, 50 ABC transporters were identified with bioinformatics-aided *in silico* analyses, and their full-length cDNA sequences were characterized. Phylogenetic analysis revealed that they could be classified into the eight subfamilies (A–H) that include all members of all ABC subfamilies. Interestingly, several teleosts’ Abcg members were closely clustered with Abch members in a distinctive clade. The *abch* gene was also observed in the coelacanth and the spotted gar, suggesting that this gene was retained from a bilaterian ancestor and that a gene loss event recently occurred in the tetrapod lineage. In teleosts, the nomenclature of previously annotated *abcg* genes should be considered carefully, as they form a distinctive clade with the marine medaka *abch* subfamily and other teleost *abch* genes, but not with the members of the Abcg subfamily.

The ATP-binding cassette (ABC) transporters are active efflux pumps that use an ATP cleavage-driven energy source for transporting endogenous and exogenous substrates (e.g., amino acids, peptides, vitamins, sugars, lipids, sterols, hormones, endogenous metabolites, inorganic anions, drugs, and metal ions) from the cytosol to the intracellular and/or extracellular region^1^,^2^. In most ABC proteins, two highly conserved structural domains, nucleotide binding domain (NBD) and/or transmembrane domain (TMD), are included in a single polypeptide possessing different numbers of each domain. Full transporters are comprised of two NBDs and two TMDs, but half transporters require homo- or heterodimerization to form a functional unit as they contain only one of each type of domain (*i.e.* NBD and TMD). Specifically, the NBDs have a responsibility to bind and hydrolyze ATP for substrate translocation across biological membranes, while the TMDs provide substrate specificity with five to six membrane-spanning helices^3^,^4^. Based on sequence similarity and phylogenetic analysis using the NBDs, the ABC transporter superfamily has been divided into eight subfamilies, A to H. Of them, the Abch subfamily was first identified in the *Drosophila melanogaster* genome and is present in sea urchin and in all of the sequenced arthropod genomes but not in fungi, plants, and mammals^5^,^6^,^7^. Interestingly, in teleosts, the *abch* gene appears only in the zebrafish (*Danio rerio*) and the green spotted pufferfish (*Tetraodon nigroviridis*)^8^ while no member of the Abch subfamily has been identified in other teleosts.

In teleosts, the tissue distribution and molecular and biochemical functions of ABC transporters were extensively reviewed, with a focus on multidrug and/or multixenobiotic resistance (MDR/MXR) and physiological properties^8^,^9^. Also, the availability of genome information for several teleosts enables us to examine the comparative evolutionary distance of the entire set of ABC transporter genes^10^. Two successive whole genome duplication (WGD) events and the additional teleost-specific genome duplication (TGD) event may have important roles in the diversification of large gene superfamilies including the ABC transporter gene families^11^,^12^. As a result, comparative evolutionary relationships of the entire ABC superfamily have been intensively examined from tetrapods to teleosts (e.g. *Danio rerio*, *Gadus morhua*, *Gasterosteus aculeatus*, *Ictalurus punctatus*, *Latimeria chalumnae*, *Oreochromis niloticus*, *Oryzias latipes*, *Takifugu rubripes*, and *Tetraodon nigroviridis*)^8^,^10^.

The marine medaka *Oryzias melastigma* (also known as Indian medaka or brackish medaka) is phylogentically close to the freshwater counterpart *Oryzias latipes* as a sister species^13^. In detail, the salinity-tolerant *O. melastigma* and *O. javanicus*, showing high adaptability in response to acute changes of the ambient osmotic pressure from freshwater to seawater, have been clustered into the same clade on phylogenetic analysis^13^,^14^. The marine medaka has increasingly been recognized as a potential model for marine environmental research, as fertilization, sperm activity, and hatchability of *O. melastigma* are feasible in freshwater and in seawater^14^. The marine medaka has several promising advantages including its small size (~3–4 cm), daily spawning, short generation time (less than 3–4 months), sexual dimorphism, responsiveness to diverse chemicals, and ease of maintenance and breeding in the laboratory^15^. Also, most of the standard experimental procedures, establishing in model fishes such as zebrafish and Japanese medaka (e.g. developmental biology using transparent embryos, semen cryopreservation and *in vitro* fertilization, cell transplantation, microinjection with DNA or RNA, primary cell culture, mutant screening, *in situ* hybridization, immunohistochemistry, and stable fluorescent transgenic lines), can be applied with slight modifications in marine medaka. To establish the marine medaka as a model animal, we obtained draft genome information with Next Generation Sequencing (NGS) technologies (unpublished data), and the information was successfully employed for the evolutionary comparison of teleosts’ gene superfamilies such as the cytochrome P450 genes^16^. Subsequently, we tried to identify the marine medaka *abch* gene, which was not annotated in the genome database of Japanese medaka, although the whole genome information of *O. latipes* is already available^17^. Interestingly, one of identified ABC members showed high similarity with the *abch* genes of zebrafish and green spotted pufferfish in our preliminary BLASTX search. Thus, our next question has been raised on the controversial suggestions of absence or presence of *abch* gene in teleosts.

In this study, we characterized 50 putative ABC transporters in *O. melastigma* and identified one putative *abch* gene. In addition, a novel phylogenetic relationship between the Abcg and Abch subfamilies in fish was uncovered and is discussed to clarify the controversial annotation of the teleost Abch family.

## Results and Discussion

### Identification and annotation of O. melastigma ABC transporters

In this study, all NBD-containing reads were examined by BLASTX searches to the non-redundant (NR) database of NCBI, and subsequently, 50 ABC transporters were identified in the genome information of *O. melastigma*. Full-length sequences of all *O. melastigma ABC* genes were characterized and registered in the GenBank database (Table 1) after annotation of each gene using *in silico* analysis (*i.e.* BLAST search, amino acid similarity and identity comparison, and domain search). The length of these full-length ABC proteins ranged from 611 amino acids (aa) (Abcf2) to 4,758 aa (Abca12). Although most *ABC* genes were annotated in *O. latipes* in the GenBank database, in-depth nomenclature and phylogenetic analysis had not been conducted in the Genus *Oryzias* as yet. Thus, this is the first report on the characterization of the entire ABC superfamily in the Genus *Oryzias*. In this paper, description and previous relevant findings on each ABC subfamily are mostly omitted as these topics were discussed thoroughly in several recent valuable reviews on fish ABC transporters^8^,^9^,^10^.

**Figure 1:**
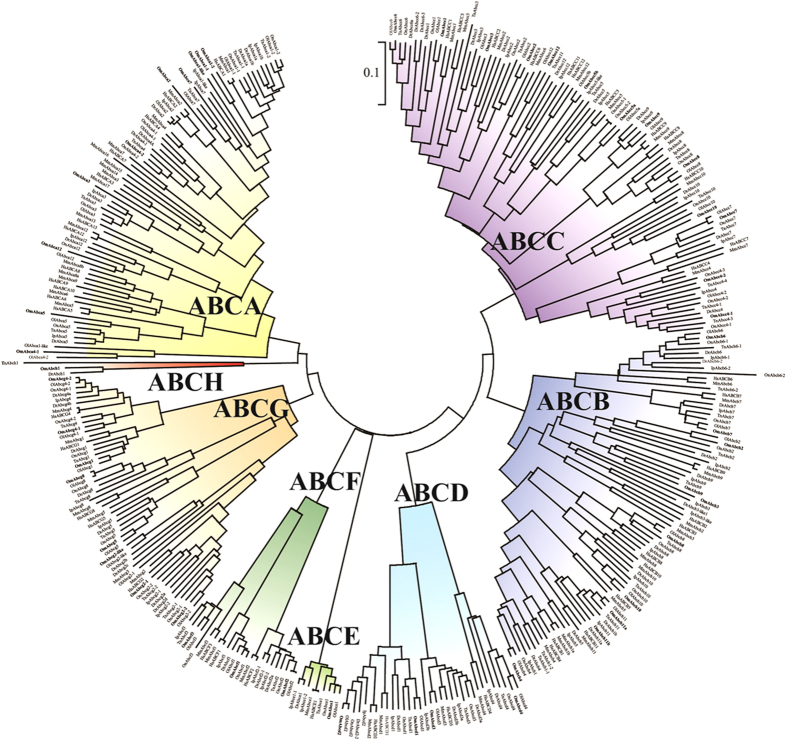
Phylogenetic analysis of 50 *O. melastigma* ABC proteins. Phylogenetic distance was calculated with combined NBD-TMD amino acid sequences from *O. melastigma* and other species. A best-fit substitution model was established using maximum likelihood (ML) analysis. Numbers at nodes represent the ML bootstrap support values and Bayesian posterior probabilities (=1.00). Details on the model test and parameters are explained in the Materials and Methods section. The tree is proportionally scaled and the scale bar indicates sequence distance in units of substitutions. Species abbreviations: Dr, *Danio rerio*; Hs, *Homo sapiens*; Ip, *Ictalurus punctatus*; Mm, *Mus musculus*; Ol, *Oryzias latipes*; Om, *Oryzias melastigma*; On, *Oreochromis niloticus*; Tn, *Tetraodon nigroviridis*.

**Figure 2:**
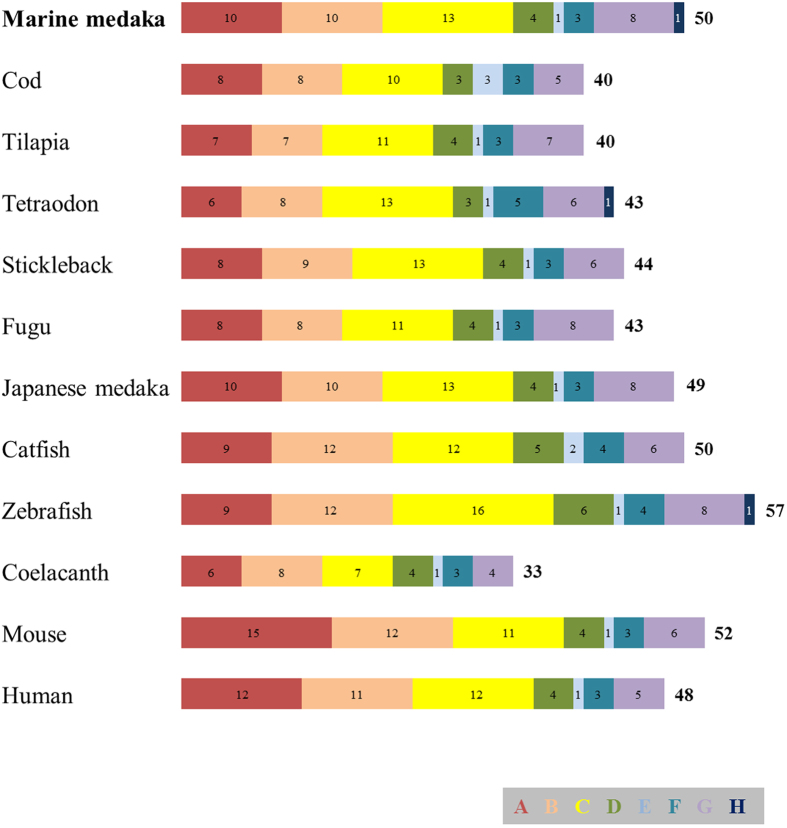
Simplified comparison of the number of genes in each subfamily of ABC transporters between *O. melastigma* and other vertebrates.

An ML phylogenetic analysis revealed that the 50 *O. melastigma* ABC transporters could be separated into the 8 subfamilies of A to H (Fig. 1). All *O. melastigma* ABC transporters possessed one or two conserved NBDs in their amino acid sequences, and the highest percentage of the ABC transporters belonged to the Abcc subfamily (26%), followed by the Abca and Abcb subfamilies (20% each) (Fig. 2). Overall composition rates and percentage rank of all *O. melastigma* ABC transporters were similar to those of other teleosts, while the absence or presence of some members belonging to each subfamily were slightly different between fish, suggesting that lineage-specific gene evolution has accumulated in teleosts (Table 2). Although overall composition of the genes seems to be conserved from teleosts to mammals, greater divergence was observed in the ABCA/Abca subfamily, as the mean number of ABCA/Abca members was higher in two mammals (*i.e.* mouse and human) compared to teleosts (Fig. 2). In this subfamily, the highest rate of gene duplication and/or loss events during evolution was suggested to be due to the absence of *abca6*, *abca8*, *abca9*, *abca10*, and *abca13* genes in teleosts^18^.

Several ABC transporters could also be divided into 26 full transporters (52%) and 20 half transporters (40%), based on the numbers of NBDs and TMDs (Fig. S1). Particularly, full transporters were observed in the Abca (10 proteins), Abcb (3 proteins), and Abcc (13 proteins) subfamilies, while half transporters were seen in the Abcb (7 proteins), Abcd (4 proteins), Abcg (8 proteins), and Abch (1 protein) subfamilies. In addition, four members involved in Abce and Abcf subfamilies that possess two linked NBDs but lack TMDs were identified in *O. melastigma*.

### Phylogenetic analysis in each ABC subfamily

To analyze the evolutionary placement of the 50 *O. melastigma ABC* genes, in-depth phylogenetic analysis was conducted for each subfamily in comparison to those of zebrafish (*D. rerio*), channel catfish (*I. punctatus*), Japanese medaka (*O. latipes*), Nile tilapia (*O. niloticus*), green spotted pufferfish (*T. nigroviridis*), mouse (*M. musculus*), and human (*H. sapiens*) (Figs S2–S7). These species were chosen to create a representative set covering general evolutionary features within the vertebrate ABC superfamily.

### Abca

Ten Abca proteins were identified in the *O. melastigma* genomic database and were examined using phylogenetic analysis to obtain their annotations (Fig. S1, Table 1). The members of the Abca subfamily of all teleosts, including marine medaka, were full transporters, as is seen in human^2^, suggesting it is a vertebrate-specific characteristic, since several insects and copepods are known to possess half transporters in this subfamily^10^,^19^. Overall phylogenetic analysis found that each member of the *O. melastigma* Abca subfamily was clustered with its counterpart from other animals. Particularly, evidence of the teleost-specific gene duplication after WGD was observed in *abca1* (*abca1-1* and *abca1–2*) and *abca4* (*abca4–1* and *abca4–2*) genes that, in mammals, are involved in cholesterol efflux of high-density lipoproteins (HDL) and the transport of retinoid-lipid complexes out of photoreceptor cells, respectively^18^,^20^,^21^. Interestingly, the *abca1-like* gene identified in catfish was also observed in both *O. melastigma* and *O. latipes* though a putative *abca1-like* gene has not been identified as yet in any other fish. Thus, the *abca1-like* gene appears to be derived from *abca1* by further genome duplication after the *abca7* duplication event in the *abca* gene lineage.

In *O. melastigma*, the mammalian-specific *ABCA6*, *ABCA8*, *ABCA9*, and *ABCA10* genes are not present in the genome, just as they are absent in other teleosts (Fig. S2). In the human genome, all these genes are located in a single cluster together with the *ABCA5* gene^18^ and in vertebrates, the ABCA5/Abca5 subfamily is highly conserved. Mammalian ABCA6/8/9/10 subfamilies form a sister clade with ABCA5 members but are not found in teleosts, indicating that the ABCA5/Abca5 lineage was split in a teleost-specific manner^5^,^10^.

### Abcb

In *O. melastigma*, there are 3 Abcb full transporters and 7 NBD-containing half transporters (Table 1), and each gene forms robust clades with their corresponding counterparts (Fig. S3). The *Abcb1*, *Abcb4*, and *Abcb5* members are known to share a common ancestor^22^, but there are controversial reports on the nomenclature for the putative *abcb1/4* gene in teleosts due to a duplication event within a range of vertebrates^10^. In detail, mammalian ABCB1 and ABCB4 arose from a lineage-specific gene duplication (Fig. S3), and this phenomenon also observed in the chicken *Gallus gallus*^8^. However, teleost *Abcb1* or *Abcb4* are not one-to-one orthologues to either ABCB1 or ABCB4 but co-orthologues to both, resulting that all teleost possesses at least one transporter in the Abcb1/Abcb4 subfamily with different annotation names (i.e. Abcb1-like, p-glycoprotein, Abcb4-like, or Abcb4)^8^, as shown in Table S1. In this study, we identified one putative *abcb4* gene in *O. melastigma* with its nomenclature. Although mammalian ABCB4 has a specific physiological function in the liver and transports certain fatty acids, a recent study showed that zebrafish Abcb4 (previously annotated as Abcb1b^5^) plays as a cellular toxicant transporter and provides protection for embryos in response to toxic chemicals^23^.

In *O. melastigma*, we could not identify the *abcb5* gene that was observed in catfish and zebrafish^10^, and no homologous sequences were found in the transcriptomes and/or genome information of Japanese medaka, Nile tilapia, and green spotted pufferfish in our *in silico* analysis. Although incomplete genomic sequence databases of these fish need to be updated in order to conduct appropriate synteny analysis, we assume that species-specific gene duplication events in Abcb5 occurred in the Abcb1 lineage. In addition, two duplicated forms of *abcb11* (*abcb11a* and *abcb11b*) appeared in marine medaka, as is also seen in Japanese medaka, zebrafish, and Atlantic cod (Ensembl Gene ID: *abcb11a*, ENSGMOG00000014190; *abcb11b*, ENSGMOG00000010088), while other teleosts have no duplicated members in the *abcb11* gene.

### Abcc

The Abcc subfamily consists of 13 full transporters in *O. melastigma* (Fig. S1, Table 1). This is the largest number of ABC transporters in all teleosts, including the marine medaka, when compared with those of mammals (Fig. 2). All members of Abcc formed distinct clades showing individual evolutionary branches (Fig. S4). In the case of *abcc4*, two types (*abcc4a* and *abcc4b*) were identified in *O. melastigma*, as is seen in other teleosts that have multiple copies of *abcc4* (two members for *O. latipes*; three members for *O. niloticus*; four members for *T. nigroviridis*), while only a single gene was identified in mammals, indicating that teleost-specific genome duplications have occurred during evolution in fish.

An interesting feature of Abcc5 divergence was observed. For example, in *O. melastigma*, two members of *abcc5* (*abcc5a* and *abcc5b*) were observed, as is seen in Japanese medaka (*abcc5a* and *abcc5b*) and catfish (*abcc5* and *abcc5*-*like*), but no additional genes were observed in tetraodon, zebrafish, or mammals (Fig. S4). Although they diverged from a common ancestor, amino acid similarity of multiple *Abcc5* members showed relatively low identity/similarity within the same group (Fig. S8, Table S2), suggesting a medaka- or catfish-specific duplication within teleosts.

A single member (*abcc12*) was incorporated into the combined clade of Abcc11/Abcc12, suggesting that the phylogenetic placement with diverse Abcc11/12 members from vertebrates requires revisiting. No member of *abcc13* was observed in the *O. melastigma* genome, as is seen in most teleosts, while a single gene of *abcc13* has been identified in zebrafish^5^.

### Abcd

The ABCD subfamily generally harbors four highly conserved members in vertebrates, while several teleosts possess the following Abcd isoforms. In *O. melastigma*, all 4 *abcd* transporters were identified as half transporters (Fig. S1, Table 1), and each *abcd* member showed a clear homologous relationship to that of vertebrates (Fig. S5). Zebrafish has two isoforms of *abcd2* and *abcd3* and catfish contains two isoforms of *abcd3*, while only a single gene has been identified in the genus *Oryzias* (*O. melastigma* and *O. latipes*), implying that *abcd* genes expanded in a lineage-specific manner in teleosts.

### Abce and Abcf

Since the subfamilies ABCE and ABCF lack TMDs and are comprised of a pair of linked NBDs, all ABC transporters are not restricted to having a function in ATP-dependent active transport. In the case of *O. melastigma* Abce and Abcf, all of the subfamily members are believed to be involved in biological processes other than transport^5^,^18^, as they possess two linked NBDs but lack TMDs (Fig. S1). Vertebrate ABCE1 was originally described as RNase L inhibitor^24^, and in recent studies novel evidences on ABCE1 roles were discovered in translation initiation, elongation, termination, and ribosome recycling^25^,^26^,^27^. The general and specific functions of vertebrate ABCF are still unclear as yet. Previously, human ABC50 (ABCF1) was identified as a tumor necrosis factor-α inducible gene in synoviocytes^28^. Subsequently, the protein was co-purified with eukaryotic translation initiation factor eIF2^29^, suggesting that it plays a role in the translation initiation at an internal ribosome entry site (IRES) *in vitro*^30^. Phylogenetic analysis revealed that all Abce and Abcf transporters formed a unique cluster, similar to those seen in vertebrates (Fig. S6). As shown in most eukaryotes, a single Abce subfamily was found in marine medaka. The Abce protein is one of the most conserved proteins as most vertebrates and all invertebrates examined so far have a single member of this group, except for catfish (two genes) and cod (three genes)^6^,^7^,^19^,^31^ (Table 2). In the case of Abcf, all Abcf homologs (*abcf1*, *abcf2*, and *abcf3*) were identified in the marine medaka and Japanese medaka genomes, while *abcf2* was duplicated in catfish and zebrafish^10^ (Fig. S6).

### Abcg

Eight members of the Abcg subfamily were identified as half transporters in *O. melastigma*, and all the Abcg members were composed with a reverse-domain architecture (NBD-TMD) (Fig. S1). In most metazoan species, this unique structural feature is common except for fungi and plants^32^,^33^. The phylogenetic analysis of *O. melastigma Abcg* supported their annotations (Fig. S7). As shown in catfish (*abcg2–1* and *abcg2–2*), green spotted pufferfish (*abcg2–1* and *abcg2–2*), and zebrafish (*abcg2a*, *abcgb*, *abcgc*, and *abcgd*), the *abcg2* gene was duplicated in the *O. melastigma* and *O. latipes* genomes (*abcg2–1*, *abcg2–2*, and *abcg2-like*). Likewise, stickleback and tilapia have 2 members and cod possesses 4 members in their genomes^8^. In particular, the *O. melastigma abcg2-like* gene was clustered with *O. latipes abcg2-like* and formed a distinct clade with zebrafish *abcg2b* and *abcg2c*. In detail, the *O. melastigma abcg2-like* gene showed low values in similarity and identity to *O. melastigma abcg2–1* and *abcg2–2* genes (Table S3). Likewise, the similarity and identity of *O. latipes abcg2-like* gene were high compared to those of the *O. melastigma abcg2-like* gene, while low similarity and identity were observed in comparison to the *O. latipes abcg2–1* and *abcg2–2* genes. Two zebrafish *abcg2* genes (*abcg2b* and *abcg2c*), clearly separated from other *abcg2* genes, show comparable patterns in similarity and identity. Thus, WGD and additional TGD may have occurred in the lineage of the teleost Abcg2 subfamily during evolution. Lineage-specific gene duplication was also observed in the Abcg4 group, as two isoforms were identified in *O. melastigma*, *O. latipes*, and *O. niloticus,* while only a single gene was observed in *I. punctatus* and *T. nigroviridis*.

### Abch

**Figure 3:**
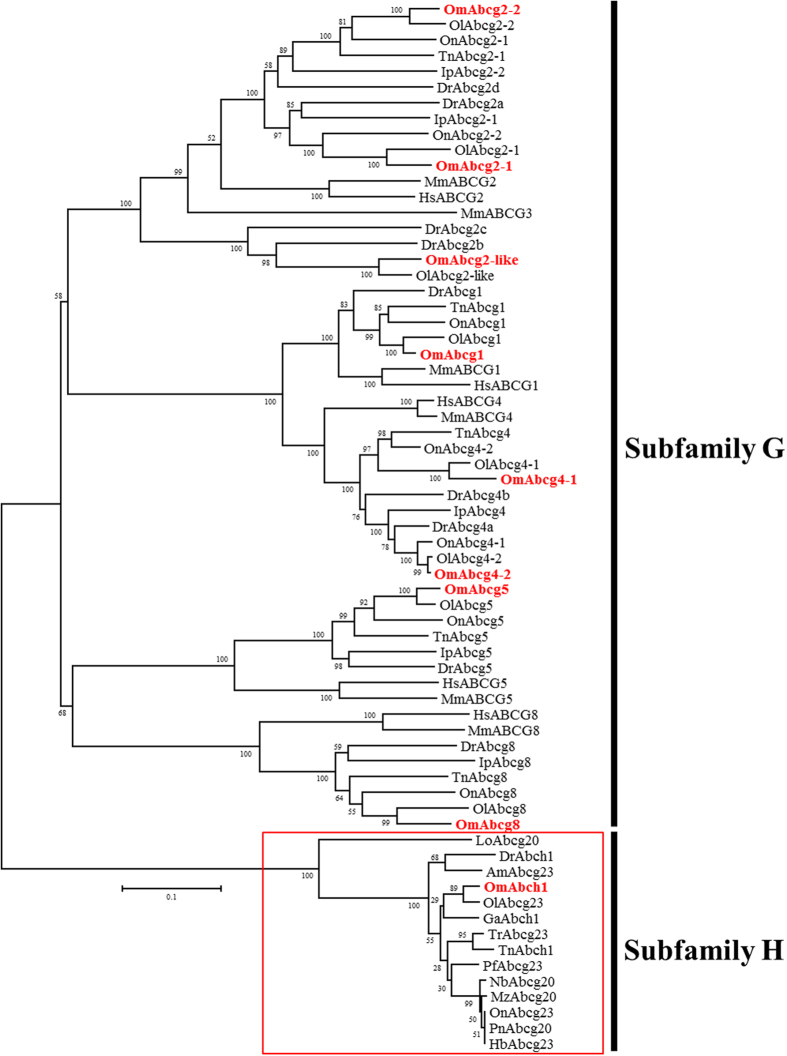
Phylogenetic analysis of Abcg/Abch transporters with additional Abch candidates. Phylogenetic distance was calculated with combined NBD-TMD amino acid sequences from *O. melastigma* and other species. A best-fit substitution model was established using maximum likelihood (ML) analysis. Numbers at nodes represent the ML bootstrap support values and Bayesian posterior probabilities. The tree is proportionally scaled, with the scale bar indicating sequence distance in units of substitutions. Species abbreviations: Am, *Astyanax mexicanus*; Dr, *Danio rerio*; Ga, *Gasterosteus aculeatus*; Hb, *Haplochromis burtoni*; Hs, *Homo sapiens*; Ip, *Ictalurus punctatus*; Lo, *Lepisosteus oculatus*; Mm, *Mus musculus*; Mz, *Maylandia zebra*; Nb, *Neolamprologus brichardi*; Ol, *Oryzias latipes*; Om, *Oryzias melastigma*; On, *Oreochromis niloticus*; Pf, *Poecilia Formosa*; Pn, *Pundamilia nyererei*; Tn, *Tetraodon nigroviridis*; Tr, *Takifugu rubripes*.

**Figure 4:**
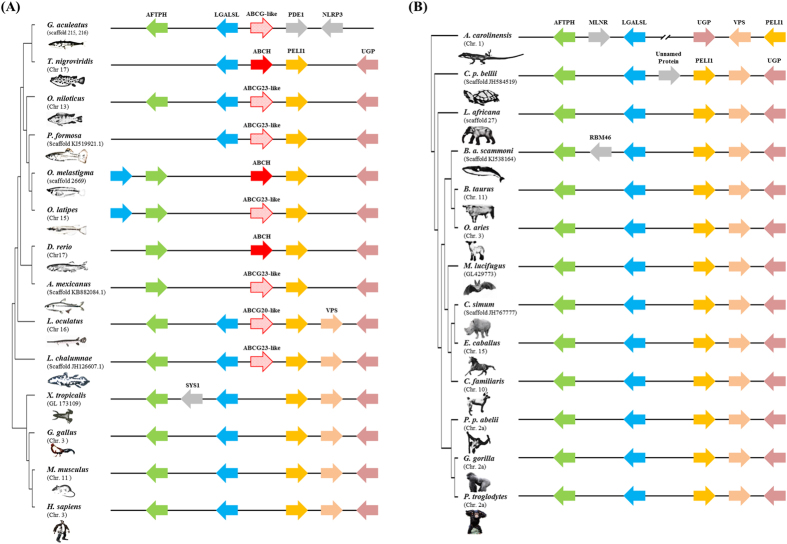
(**A**) Syntenic analysis of the genomic structure harboring the abch gene in teleosts. Genes are represented by colored arrows. Distances within genes are not represented to scale. The different species were drawn by author Chang-Bum Jeong using Photoshop. GenBank accession numbers of ABCG/Abcg members used in the syntenic analysis are as follows: Astyanax mexicanus Abcg23-like (cavefish; XP_007244688), Gasterosteus aculeatus Abcg-like (Stickleback; ENSGACG00000000159), Latimeria chalumnae Abcg23-like (coelacanth; XP_005989631), Lepisosteus oculatus Abcg20-like (spotted gar; XP_006638801), Oreochromis niloticus Abcg23-like (tilapia; XP_005473794), Oryzias latipes Abcg23-like (Japanese medaka; XP_011482890), and Poecilia formosa Abcg23-like (Amazon molly; XP_007572984). Gene name abbreviations: aftiphilin, AFTPH; galectin-related protein, LGALSL; NACHT, LRR and PYD domain-containing protein, NLRP3; E3 ubiquitin-protein ligase, PELI1; calmodulin-dependent phosphodiesterase 1C, PDE1; golgi-localized integral membrane protein, SYS1; UTP-glucose-1-phosphate uridylyltransferase, UGP; vacuolar protein sorting-associated protein, VPS. (**B**) Further syntenic analysis of the genomic structure of abch gene in tetrapods. Genes are represented by colored arrows. Distances within genes are not represented to scale. Entire images were drawn by the author Chang-Bum Jeong. Gene name abbreviations: aftiphilin, AFTPH; galectin-related protein, galectin-related protein, LGALSL; E3 ubiquitin-protein ligase, PELI1; motilin receptor, MLNR; probable RNA-binding protein 46 isoform 2, RBM46; UTP-glucose-1-phosphate uridylyltransferase, UGP; vacuolar protein sorting-associated protein, VPS.

**Figure 5:**
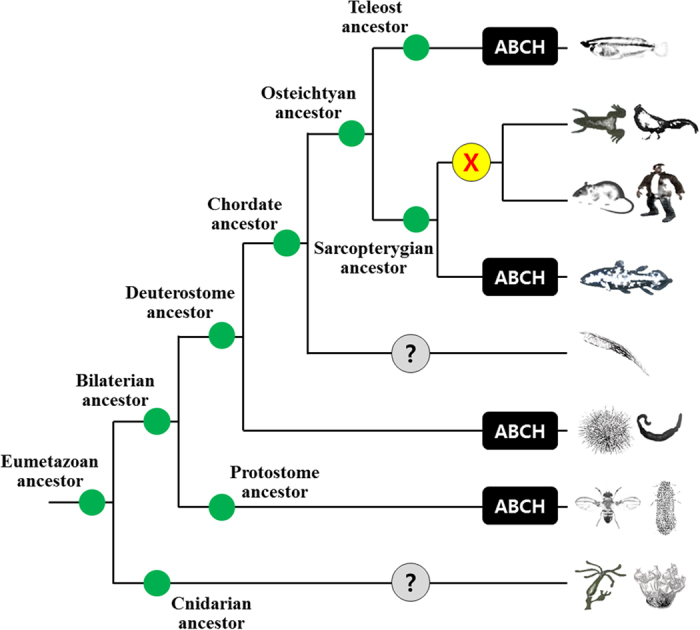
Schematic diagram for evolutionary scenario of the abch gene in eumetazoans. The diagram depicts a phylogenetic tree of metazoan species. Gene loss event is marked with an “X” in a yellow circle. Unknown information on the existence of the abch gene is marked with a “?” in a gray circle. The different species were drawn by the author Chang-Bum Jeong using Photoshop. GenBank accession numbers of ABCG genes used in the syntenic analysis are as follows: Drosophila melanogaster CG9990, CG11147, and CG33970 (fruitfly; AAF56807, AAF52284, and ABC66191, respectively); Latimeria chalumnae abch (coelacanth; ENSLACG00000015557); Oryzias melastigma abch (marine medaka; KP725053); Saccoglossus kowalevskii abch (acorn worm; XP_006817503); Tribolium castaneum abch1, abch2, and abch3 (red flour beetle; XP_973444, XP_967359, and XP_974932, respectively).

To date, the *abch* genes have only been annotated in sea urchin, arthropods, and two teleosts, zebrafish and green spotted pufferfish^5^,^6^,^7^,^8^,^18^,^19^. In fact, the presence of the Abch subfamily in fish is controversial. Previously, the *abch* gene was annotated only in zebrafish and a putative form was identified in green spotted pufferfish^34^, which has been confirmed in a recent review of ABC drug transporters^8^. The Abch subfamily has not been identified in other fishes such as catfish, Japanese medaka, fugu, stickleback, tetraodon, tilapia, cod, or coelacanths^10^. Interestingly, in *O. melastigma*, one transporter formed the same clade with the *abch* genes of zebrafish (*abch1*) and green spotted pufferfish (*abch1*), but this Abch clade seems to be an outgroup of the Abcg subfamily (Fig. S7). To clarify the identity of the Abch group, several Abcg members (*abcg20* to *abcg23*) from other fish that contained only partial ABC information were employed in the phylogenetic analysis (Fig. 3). To date, no information on the annotation or phylogenetic distance of these Abcg members has been found. Regardless of the absence of the information for the Abch clade, our phylogenetic analysis strongly supported the formation of a unique clade for the three Abch members and additional Abcg20/Abcg23 members. In this study, we supposed that *O. melastigma* has the Abch subfamily, although the distinct clade containing Abch members and some Abcg members were duplicated from the Abcg subfamily. An independent lineage-specific expansion likely induced this kind of unique evolutionary event, as the Abch subfamily was not identified in coelacanths, the most primitive fish. A synteny comparison supported the orthologous relationship, as the genomic structures of the neighboring genes were very similar among teleosts (Fig. 4A). Interestingly, the *abch* gene was conserved in the coelacanth *Latimeria chalumnae* and the spotted gar *Lepisosteous oculatus* genomes, while there is no *Abch* orthologue in tetrapods (Fig. 4B). These results suggest that the *abch* gene was retained in the ancestor of teleosts and sarcopterygian. In fact, the *abch* genes are conserved in nematodes, copepods, and insects^6^,^7^,^10^,^19^,^31^ and observed in the Pacific oyster *Crassostrea gigas* (EKC37771), the acorn worm *Saccoglossus kowalevskii* (XP_006817503), and the sea urchin *Strongylocentrotus purpuratus* (XP_003731550; also registered in the Sea urchin genome database (http://www.echinobase.org); ID: SPU_026438) by GenBank database search, suggesting that the *abch* gene was retained from the bilaterian ancestor and a gene loss event recently occurred in the tetrapod lineage (Fig. 5). In the GenBank database, additional sea urchin ABC transporter H family member 2-like (*S. purpuratus*; LOC100889169), the dolphin ABC transporter H family member 2-like (*Lipotes vexillifer*; LOC103069646), and the Tibetan antelope ABC transporter H family member 2-like (*Pantholops hodgsonii*; LOC102331264) were registered as potential ABCH/Abch members, but they formed a distinct phylogenetic clade between Abcg and Abcf subfamilies apart from the Abch subfamily (Fig. S9, S11). Thus, the gene loss event of Abch subfamily in the tetrapod lineage is robust based on this result. At the moment, no putative *abch* gene has been identified in the Florida lancelet (*Branchiostoma floridae*) and Cnidarian. Identification and characterization of additional metazoan *abch* genes would be helpful for a better understanding of Abch evolution. Taken together, the nomenclature of several members of the Abcg subfamily that cluster with Abch should be reconsidered, as this may be an independent subfamily. This information provides a resource as a whole set from an essential gene family in teleosts, and their phylogenetic relationships with synteny analysis will be useful for a better understanding of evolutionary aspects of the ABC superfamily in teleosts.

## Materials And Methods

### Ethics in experiments

All animal handling and experimental procedures were approved by the Animal Welfare Ethical Committee and the Animal Experimental Ethics Committee of the Sungkyunkwan University (Suwon, South Korea).

### Fish

The marine medaka *O. melastigma* were kindly provided by Dr. Doris W.T. Au (City University of Hong Kong, Hong Kong SAR, China) and were maintained at the aquarium facility of the Department of Biological Science, Sungkyunkwan University (Suwon, South Korea). The fish were reared in accordance with the Animal Welfare Ethical Committee of the Sungkyunkwan University. Briefly, the fish were maintained in automatically controlled conditions at 26°C with a light/dark ratio of 12L:12D and artificial seawater (TetraMarine Salt Pro, Tetra™, Cincinnati, OH, USA; 5.71 ± 0.19 mgO_2_/L) adjusted to 12 practical salinity units (psu). The automated water-changing system was set for constant flow-through, and water quality (pH, salinity, and temperature) was recorded using various instruments. Fish were maintained in glass aquaria (60 L capacity) and each aquarium accommodated up to 30 adult fish (both sexes). They were fed *Artemia salina* (<24 h after hatching) once a day until satiation.

### Retrieval of ABC transporter genes

An *O. melastigma* genomic DNA database was constructed using next generation sequencing (NGS) technologies and bioinformatics (unpublished data; scaffold no.: 24,820; total length: 671,972,662 bp; longest length: 1,068,498 bp; average length: 27,074 bp; N50 value: 115,707 bp). Briefly, the genomic DNA of *O. melastigma* was mechanically sheared into fragments, and a genomic DNA library was created according to the manufacturer’s instructions (Roche Applied Science, Genome Sequencer System, Pleasanton, CA, USA). Genomic DNA of *O. melastigma* was sequenced using a GS-FLX-Titanium genomic DNA sequencer (Roche Diagnostics, Mannheim, Germany) and SOLEXA sequencer (Illumina, San Diego, CA, USA), and then properly assembled with the software NGS Cell (Ver. 4.06 beta 67189, CLC Bio, Boston, MA, USA) and Velvet (EMBL-EBI, Wellcome Trust Genome Campus, Cambridge, UK). Currently, the number of unidentified N in the consensus sequence is 50,688,736 bp, indicating that approximately 7.5% of nucleotides are unidentified. To date, we have obtained 46,461 genes (unpublished data; total length: 27,691,766 bp; longest length: 7,011 bp; N50 value: 2,265 bp) after *de novo* genome assembly and RNA-sequencing (contig no.: 51,014; total length of contigs: 135,850,078 bp; longest length among contigs: 21,254 bp) with bioinformatics-aided gene annotation.

To obtain the sequence information for all ABC transporters after assembly, all contigs containing NBDs and/or TMDs in *O. melastigma* genomic DNA and transcriptome databases were subjected to BLAST analysis using the non-redundant (NR) database at GenBank (http://www.ncbi.nlm.nih.gov/genome/seq/database.html).

### Nucleotide sequence validation

*O. melastigma* pooled tissues were homogenized in three volumes of TRIZOL^®^ reagent (Invitrogen, Paisley, Scotland) with a tissue grinder and stored at −80 °C until use. Total RNA was extracted according to the manufacturers’ instructions and stored at −80 °C until use. DNA digestion was performed using DNase I (Sigma, St. Louis, MO, USA). Total RNA was quantified by absorption of light at 230, 260, and 280 nm (A230/260, A260/280) using a spectrophotometer (Qiaxpert®, Qiagen, Hilden, Germany). To verify that there was no genomic DNA contamination, total RNAs were loaded in a 1% agarose gel that contained ethidium bromide (EtBr) and visualized on a UV transilluminator (Wealtec Corp., Sparks, NV, USA). Subsequently, total RNAs were loaded in a 1% formaldehyde/agarose gel with EtBr staining in order to verify total RNA quality and verify *18/28S* ribosomal RNA integrity. After RNA quality was determined, single-stranded cDNA was synthesized from 2 μg of total RNA from each sample using oligo (dT)_20_ primers for reverse transcription in 20 μl reactions (SuperScript^TM^ III RT kit, Invitrogen).

To confirm exon/intron boundaries and start/stop codons of *O. melastigma* ABC transporter genes, genomic structures of the obtained genes were compared between genomic clones and the transcripts for each gene. Some incomplete ABC transporter sequences were subjected to 5′- and 3′-RACE according to the manufacturer’s protocol (Invitrogen, Carlsbad, CA, USA). To validate cDNA sequences of entire *ABC* genes identified in *O. melastigma*, RT-PCR was employed with two primers: a forward primer containing a start codon, and a reverse primer containing a stop codon. RT-PCR was conducted in a reaction mixture comprising 1 μl of first strand cDNA, 5 μl of 10× PCR reaction buffer, 1 μl of 10 mM dNTPs, 10 pM concentrations of each primer, and 0.5 μl of NeoTherm™ Taq polymerase (GeneCraft, Köln, Germany). Reaction mixtures were subjected to amplification (1 cycle, 95 °C, 5 min; 30 cycles, 94 °C, 30 sec, 55 °C, 30 sec, and 72 °C, 30 sec; 1 cycle, 72 °C, 7 min) using an iCycler (Bio-Rad, Hercules, CA, USA). The final PCR products were isolated from 1% agarose/Tris-Borate-EDTA (TBE) gels, cloned into pCR2.1 TA vectors (Invitrogen), and sequenced using an ABI PRISM 3700 DNA analyzer (Bionics Co., Seoul, South Korea).

### Annotation of whole ABC transporter genes

Annotation and nomenclature of all the *O. melastigma ABC* genes followed amino acid sequence similarities compared to ABC superfamilies of other animals in terms of *in silico* domain analysis [Pfam HMM search, http://pfam.sanger.ac.uk; Motif Scan, http://myhits.isb-sib.ch/cgi-bin/motif_scan; National Center for Biotechnology Information (NCBI)’s Conserved Domain Database (CDD)]. All gene information was registered with the GenBank database, and the accession numbers of each gene are appended in Table 1.

### Phylogenetic analysis

To investigate evolutionary relationships and the nomenclature of *O. melastigma* putative ABC transporters, amino acid sequences of ABC transporters identified in *O. melastigma* were subjected to phylogenetic analysis and compared with other species (*Danio rerio*, *Ictalurus punctatus*, *Oreochromis niloticus*, *Oryzias latipes*, *Tetraodon nigroviridis*, *Mus musculus*, and *Homo sapiens*) in the NCBI GenBank database by performing BLASTX searches. Combined NBD-TMD amino acid sequences from *O. melastigma* and other species were aligned using MEGA software (ver. 6.0; Center for Evolutionary Medicine and Informatics, Tempe, AZ, USA) with the ClustalW alignment algorithm. To set a best-fit substitution model for phylogenetic analysis, a model showing the lowest score in the Bayesian Information Criterion (BIC)^35^ and the Akaike Information Criterion (AICc)^36^,^37^ was determined with Maximum Likelihood (ML) analysis. According to the results of the model test, the WAG + G + I + F model was chosen to generate a phylogenetic tree. MrBayes (ver. 3.1.2) was used to reconstruct phylogenetic trees based on Bayesian inference^38^. The Markov chain Monte Carlo (MCMC) process was conducted with four chains and run for 5,000,000 generations. Sampling frequency was every 100 generations. After analysis, the first 5,000 trees were deleted as part of the burn-in process. A consensus tree was constructed and was visualized using SeaView ver.4.2.1^39^. Nodal support was reported as Bayesian posterior probabilities (=1.00).

## Additional Information

**How to cite this article**: Jeong, C.-B. *et al.* Marine medaka ATP-binding cassette (ABC) superfamily and new insight into teleost Abch nomenclature. *Sci. Rep.* **5**, 15409; doi: 10.1038/srep15409 (2015).

## Supplementary Material

Supplementary Information

## Figures and Tables

**Table 1:** 50 ABC transporters identified in the marine medaka *O. melastigma* genome.

Gene	Size (AA)	Topology	Accession No.	Matched gene	Matched species	E-value
*abca1-1*	2295	TM-NBD-TM-ABC	KP725006	*abca1* (XP_004086583)	*Oryzias latipes*	0.0
*abca1-2*	2271	TM-NBD-TM-ABC	KP725007	*abca1* (XP_004066316)	*Oryzias latipes*	0.0
*abca1-like*	2073	TM-NBD-TM-ABC	KP725008	*abca1* (XP_004554555)	*Maylandia zebra*	0.0
*abca2*	2514	TM-NBD-TM-ABC	KP725010	*abca2* (XP_005447927)	*Oreochromis niloticus*	0.0
*abca3*	1707	TM-NBD-TM-ABC	KP725009	*abca3* (XP_004071707)	*Oryzias latipes*	0.0
*abca4-1*	2275	TM-NBD-TM-ABC	KP725013	*abca4* (XP_004080989)	*Oryzias latipes*	0.0
*abca4-2*	2312	TM-NBD-TM-ABC	KP725014	*abca4* (XP_005476689)	*Oreochromis niloticus*	0.0
*abca5*	1687	TM-NBD-TM-ABC	KP725005	*abca5* (XP_004080721)	*Oryzias latipes*	0.0
*abca7*	2353	TM-NBD-TM-ABC	KP725011	*abca1* (XP_004068024)	*Oryzias latipes*	0.0
*abca12*	4758	TM-NBD-TM-ABC	KP725012	*abca12* (XP_003445395)	*Oreochromis niloticus*	0.0
*abcb2*	732	TM-NBD	KP725027	*abcb2* (XP_004078551)	*Oryzias latipes*	0.0
*abcb3*	713	TM-NBD	KP725050	*abcb3* (XP_004080734)	*Oryzias latipes*	0.0
*abcb4*	1290	TM-NBD-TM-ABC	KP725016	*abcb1* (ADQ20481)	*Poeciliopsis lucida*	0.0
*abcb6*	850	TM-NBD	KP725020	*abcb6* (XP_004066768)	*Oryzias latipes*	0.0
*abcb7*	746	TM-NBD	KP725018	*abcb7* (XP_004073395)	*Oryzias latipes*	0.0
*abcb8*	716	TM-NBD	KP725026	*abcb8* (XP_004079446)	*Oryzias latipes*	0.0
*abcb9*	806	TM-NBD	KP725040	*abcb9* (XP_005806663)	*Xiphophorus maculatus*	0.0
*abcb10*	701	TM-NBD	KP725031	*abcb10* (XP_004067419)	*Oryzias latipes*	0.0
*abcb11a*	1364	TM-NBD-TM-ABC	KP725033	*abcb11* (XP_004066603)	*Oryzias latipes*	0.0
*abcb11b*	1306	TM-NBD-TM-ABC	KP725019	*abcb11* (XP_004081955)	*Oryzias latipes*	0.0
*abcc1*	1511	TM-NBD-TM-ABC	KP725025	*abcc1* (XP_004573179)	*Maylandia zebra*	0.0
*abcc2*	1567	TM-NBD-TM-ABC	KP725035	*abcc2* (XP_004077201)	*Oryzias latipes*	0.0
*abcc3*	1543	TM-NBD-TM-ABC	KP725036	*abcc3* (XP_004086831)	*Oryzias latipes*	0.0
*abcc4-1*	1324	TM-NBD-TM-ABC	KP725032	*abcc4* (XP_005744288)	*Pundamilia nyererei*	0.0
*abcc4-2*	1329	TM-NBD-TM-ABC	KP725029	*abcc4* (XP_005945197)	*Haplochromis burtoni*	0.0
*abcc5a*	1383	TM-NBD-TM-ABC	KP725030	*abcc5* (XP_005751691)	*Pundamilia nyererei*	0.0
*abcc5b*	1384	TM-NBD-TM-ABC	KP725039	*abcc5* (XP_004073112)	*Oryzias latipes*	0.0
*abcc6*	1510	TM-NBD-TM-ABC	KP725034	*abcc6* (XP_004066300)	*Oryzias latipes*	0.0
*abcc7*	1502	TM-NBD-TM-ABC	KP725028	*abcc7* (AFV39711)	*Oryzias dancena*	0.0
*abcc8*	1587	TM-NBD-TM-ABC	KP725041	*abcc8* (XP_005803356)	*Xiphophorus maculatus*	0.0
*abcc9*	1568	TM-NBD-TM-ABC	KP725042	*abcc9* (XP_004083272)	*Oryzias latipes*	0.0
*abcc10*	1535	TM-NBD-TM-ABC	KP725043	*abcc7* (XP_004065626)	*Oryzias latipes*	0.0
*abcc12*	1368	TM-NBD-TM-ABC	KP725038	*abcc9* (XP_004067574)	*Oryzias latipes*	0.0
*abcd1*	772	TM-NBD	KP725046	*abcd1* (XP_004086440)	*Oryzias latipes*	0.0
*abcd2*	734	TM-NBD	KP725048	*abcd2* (XP_004082884)	*Oryzias latipes*	0.0
*abcd3*	657	TM-NBD	KP725044	*abcd3* (XP_004080884)	*Oryzias latipes*	0.0
*abcd4*	616	TM-NBD	KP725045	*abcd4* (XP_004083921)	*Oryzias latipes*	0.0
*abce1*	599	NBD-NBD	KP725037	*abce1* (XP_004079653)	*Oryzias latipes*	0.0
*abcf1*	819	NBD-NBD	KP725049	*abcf1* (XP_004074386)	*Oryzias latipes*	0.0
*abcf2*	611	NBD-NBD	KP725051	*abcf2* (XP_004081198)	*Oryzias latipes*	0.0
*abcf3*	711	NBD-NBD	KP725047	*abcf3* (XP_003457877)	*Oreochromis niloticus*	0.0
*abcg1*	670	NBD-TM	KP725015	*abcg1* (XP_004086643)	*Oryzias latipes*	0.0
*abcg2-1*	652	NBD-TM	KP725017	*abcg2* (XP_004079651)	*Oryzias latipes*	0.0
*abcg2-2*	664	NBD-TM	KP725054	*abcg2* (XP_006787117)	*Neolamprologus brichardi*	0.0
*abcg2-like*	616	NBD-TM	KP725024	*abcg2* (XP_004066087)	*Oryzias latipes*	0.0
*abcg4-1*	648	NBD-TM	KP725023	*abcg4* (XP_004076001)	*Oryzias latipes*	0.0
*abcg4-2*	642	NBD-TM	KP725022	*abcg4* (XP_004076385)	*Oryzias latipes*	0.0
*abcg5*	638	NBD-TM	KP725021	*abcg5* (XP_004077344)	*Oryzias latipes*	0.0
*abcg8*	666	NBD-TM	KP725052	*abcg8* (XP_003438483)	*Oreochromis niloticus*	0.0
*abch*	700	NBD-TM	KP725053	*abcg23-like* (XP_011482890)	*Oryzias latipes*	0.0

**Table 2:** Number of ABC transporters in vertebrates and the composition of each ABC subfamily.

Gene	Human	Mouse	Catfish	Zebrafish	Japanese Medaka	Marine Medaka	Fugu	Stickleback	Tetraodon	Tilapia	Cod	Coelacanth
ABCA1	1	1	3	2	3	**3**	2	2	2	2	2	1
ABCA2	1	1	1	1	1	**1**	0	0	0	0	0	0
ABCA3	1	1	1	1	1	**1**	1	1	1	1	1	1
ABCA4	1	1	1	2	2	**2**	2	2	1	2	2	1
ABCA5	1	1	1	1	1	**1**	1	1	1	1	1	1
ABCA6	1	1	0	0	0	**0**	0	0	0	0	0	0
ABCA7	1	1	1	1	1	**1**	1	1	1	0	1	1
ABCA8	1	2	0	0	0	**0**	0	0	0	0	0	0
ABCA9	1	1	0	0	0	**0**	0	0	0	0	0	0
ABCA10	1	0	0	0	0	**0**	0	0	0	0	0	0
ABCA12	1	1	1	1	1	**1**	1	1	0	1	1	1
ABCA13	1	1	0	0	0	**0**	0	0	0	0	0	0
ABCA14	0	1	0	0	0	**0**	0	0	0	0	0	0
ABCA15	0	1	0	0	0	**0**	0	0	0	0	0	0
ABCA17	0	1	0	0	0	**0**	0	0	0	0	0	0
ABCB1	1	2	1	1	0	**0**	0	1	0	0	1	0
ABCB2	1	1	1	1	1	**1**	1	1	1	1	1	1
ABCB3	1	1	2	1	1	**1**	0	0	0	0	0	0
ABCB4	1	1	0	0	1	**1**	0	0	0	1	0	0
ABCB5	1	1	1	1	0	**0**	0	0	0	0	0	1
ABCB6	1	1	2	2	1	**1**	2	2	2	2	1	1
ABCB7	1	1	1	1	1	**1**	1	1	1	1	1	1
ABCB8	1	1	1	1	1	**1**	1	1	1	1	1	1
ABCB9	1	1	1	1	1	**1**	1	1	1	0	1	1
ABCB10	1	1	1	1	1	**1**	1	1	1	1	1	1
ABCB11	1	1	1	2	2	**2**	1	1	1	0	1	1
ABCC1	1	1	1	1	1	**1**	1	1	1	1	0	1
ABCC2	1	1	1	1	1	**1**	1	1	1	1	1	1
ABCC3	1	1	1	1	1	**1**	1	1	1	1	0	0
ABCC4	1	1	1	1	2	**2**	3	4	4	3	3	1
ABCC5	1	1	2	1	2	**2**	1	1	1	2	1	0
ABCC6	1	1	1	3	1	**1**	1	2	2	1	1	1
ABCC7	1	1	1	1	1	**1**	0	0	0	0	1	0
ABCC8	1	1	1	3	1	**1**	1	1	1	1	1	1
ABCC9	1	1	1	1	1	**1**	0	0	0	0	0	1
ABCC10	1	1	1	1	1	**1**	1	1	1	1	1	1
ABCC11	1	0	0	0	0	**0**	0	0	0	0	0	0
ABCC12	1	1	1	1	1	**1**	1	1	1	0	1	0
ABCC13	0	0	0	1	0	**0**	0	0	0	0	0	0
ABCD1	1	1	1	1	1	**1**	1	1	1	1	0	1
ABCD2	1	1	1	2	1	**1**	1	1	0	1	1	1
ABCD3	1	1	2	2	1	**1**	1	1	1	1	1	1
ABCD4	1	1	1	1	1	**1**	1	1	1	1	1	1
ABCE1	1	1	2	1	1	**1**	1	1	1	1	3	1
ABCF1	1	1	1	1	1	**1**	1	1	3	1	1	1
ABCF2	1	1	2	2	1	**1**	1	1	1	1	1	1
ABCF3	1	1	1	1	1	**1**	1	1	1	1	1	1
ABCG1	1	1	1	1	1	**1**	1	0	0	1	0	1
ABCG2	1	1	2	3	3	**3**	2	2	2	2	2	0
ABCG3	0	1	0	0	0	**0**	0	0	0	0	0	0
ABCG4	1	1	1	2	2	**2**	3	2	2	2	1	1
ABCG5	1	1	1	1	1	**1**	1	1	1	1	1	1
ABCG8	1	1	1	1	1	**1**	1	1	1	1	1	1
ABCH	0	0	0	1	0	**1**	0	0	1	0	0	0
ABCH (updated)	**0**	**0**	**?**	**1**	**1**	**1**	**1**	**1**	**1**	**1**	**1**	**1**
Total	48	52	50	57	50	**50**	44	45	43	41	41	34

This table was updated from Liu *et al.*^10^. In detail, the *abch* gene was recently confirmed in the green spotted pufferfish (*Tetraodon nigroviridis*)^8^ . In this study, additional 12 ABC transporter members were newly identified in the genome database (http://genome.ucsc.edu) of Japanese medaka (*Oryzias latipes*), although Liu *et al.*^10^ reported 38 ABC transporters in the fish.
