# Supplementary material for: Marine medaka ATP-binding cassette (ABC) superfamily and new insight into teleost Abch nomenclature
Source: Sci Rep. 2015 Oct 16;5:15409. doi: 10.1038/srep15409 (PMC4607974; doi:10.1038/srep15409)
Supplement: Supplementary Information [file srep15409-s1.pdf]

Supplementary file for:

Marine medaka ATP-binding cassette (ABC) superfamily and new insight into teleost Abch nomenclature

Chang-Bum Jeong[a,b,#], Bo-Mi Kim[a,#], Hye-Min Kang[a], Ik-Young Choi[c], Jae-Sung Rhee[d,*], Jae-Seong Lee[a,*]

[a] *Department of Biological Science, College of Science, Sungkyunkwan University, Suwon 440-746, South Korea*

[b] *Department of Chemistry, College of Natural Sciences, Hanyang University, Seoul 133-791, South Korea*

[c] *National Instrumentation Center for Environmental Management, College of Agriculture and Life Sciences, Seoul National University, Seoul 151-921, South Korea*

[d] *Department of Marine Science, College of Natural Sciences, Incheon National University, Incheon 406-772, South Korea*

[#]These authors contributed equally to this manuscript.

*Corresponding authors: jslee2@skku.edu (J.-S. Lee[a]), jsrhee@inu.ac.kr (J.-S. Rhee[d])

Table S1. Information on the putative *abcb1/4* genes in teleosts registered in GenBank database and/or Ensembl genome browser.

Species	Gene name	Ensembl Gene ID	GenBank No.
Astyanax mexicanus	Abcb4	ENSAMXG00000016165	
Danio rerio	Abcb4	ENSDARG00000010936	AFR69055
Gadus morhua	Abcb4	ENSGMOG00000015175	
Gasterosteus aculeatus	Abcb4	ENSGACP00000012286	
Ictalurus punctatus	Abcb1		JT312655
Latimeria chalumnae	Abcb4	ENSLACG00000008552	
Lepisosteus oculatus	Abcb4	ENSLOCG00000010373	
Oncorhynchus mykiss	Abcb1		AAW56424
Oreochromis niloticus[1]	P-glycoprotein<br>Abcb4	 ENSONIP00000007731	BAM36701
Oryzias latipes[2]	Abcb1<br>Abcb4	 ENSORLP00000011622	101171435
Petromyzon marinus[3]	Abcb1<br>Abcb4	 ENSPMAG00000009053	AKC42134
Poecilia formosa	Abcb4	ENSPFOG00000016540	
Poeciliopsis lucida	Abcb1		ADQ20481
Scleropages formosus	Abcb4		KKX05686
Takifugu rubripes	Abcb4	ENSTRUG00000005770	
Tetraodon nigroviridis[4]	Abcb4-1<br>Abcb4-2<br>Abcb4-3	ENSTNIG00000018356<br>ENSTNIG00000018357<br>ENSTNIG00000018358	CAG11904<br>CAG11905<br>CAG11906
Trematomus bernacchii	Abcb1		ACX30417
Xiphophorus maculatus	Abcb4	ENSXMAG00000006743	

[1]A single gene annotated with different names.

[2]A single gene annotated with different names.

[3]A single gene annotated with different names.

[4]Three genes annotated individually.

Table S2. Amino acid similarity and identity comparisons for *Oryzias melastigma* and *Ictalurus punctatus abcc5* genes, which are separated into distinctive clades. Abbreviations: Om, *Oryzias melastigma*; Ip, *Ictalurus punctatus*.

		**Similarity (%)**			
		OmAbcc5a	OmAbcc5b	IpAbcc5	IpAbcc5-like
**Identity (%)**	OmAbcc5a	1.00	0.66	0.78	0.66
	OmAbcc5b	0.55	1.00	0.68	0.81
	IpAbcc5	0.71	0.56	1.00	0.68
	IpAbcc5-like	0.55	0.71	0.57	1.00

Table S3. Amino acid similarity and identity comparisons for *Oryzias melastigma*, *Oryzias latipes*, and *Danio rerio abcg2* genes, which are separated into distinctive clades. Abbreviations: Om, *Oryzias melastigma*; Ol, *Oryzias latipes*; Dr, *Danio rerio*.

		**Similarity (%)**									
		OmAbcg2-1	OmAbcg2-2	OmAbcg2-like	OlAbcg2-1	OlAbcg2-2	OlAbcg2-like	DrAbcg2a	DrAbcg2b	DrAbcg2c	DrAbcg2d
**Identity (%)**	OmAbcg2-1	1.00	0.76	0.60	0.92	0.75	0.60	0.81	0.60	0.59	0.68
	OmAbcg2-2	0.66	1.00	0.59	0.72	0.95	0.59	0.76	0.59	0.58	0.69
	OmAbcg2-like	0.48	0.46	1.00	0.57	0.58	0.95	0.61	0.81	0.77	0.54
	OlAbcg2-1	0.89	0.63	0.45	1.00	0.71	0.57	0.76	0.56	0.55	0.64
	OlAbcg2-2	0.65	0.93	0.46	0.62	1.00	0.58	0.74	0.59	0.57	0.68
	OlAbcg2-like	0.48	0.93	0.93	0.45	0.46	1.00	0.61	0.83	0.77	0.54
	DrAbcg2a	0.74	0.66	0.48	0.69	0.64	0.48	1.00	0.60	0.60	0.68
	DrAbcg2b	0.48	0.46	0.73	0.44	0.46	0.75	0.48	1.00	0.74	0.54
	DrAbcg2c	0.48	0.46	0.68	0.44	0.45	0.69	0.48	0.66	1.00	0.53
	DrAbcg2d	0.58	0.61	0.43	0.55	0.60	0.44	0.58	0.43	0.42	1.00

Supplementary Figure Legends

Figure S1. Schematic representation of the conserved domain analysis of 50 *O. melastigma* ABC transporters. NBD in an ellipse indicates nucleotide binding domains (also referred to as an ATP-binding cassette), and TM in a rectangle represents transmembrane domains (TMDs). Numbers in brackets refer to the number of ABC proteins in each subfamily.

Figure S2. Phylogenetic analysis of the *O. melastigma* Abca subfamily, compared with those of other species, using the Bayesian method. Numbers at branch nodes represent the confidence level of posterior probability. Species abbreviations: Dr, *Danio rerio*; Hs, *Homo sapiens*; Ip, *Ictalurus punctatus*; Mm, *Mus musculus*; Ol, *Oryzias latipes*; Om, *Oryzias melastigma*; On, *Oreochromis niloticus*; Tn, *Tetraodon nigroviridis*.

Figure S3. Phylogenetic analysis of the *O. melastigma* Abcb subfamily, compared with those of other species, using the Bayesian method. Numbers at branch nodes represent the confidence level of posterior probability. Dr, *Danio rerio*; Hs, *Homo sapiens*; Ip, *Ictalurus punctatus*; Mm, *Mus musculus*; Ol, *Oryzias latipes*; Om, *Oryzias melastigma*; On, *Oreochromis niloticus*; Tn, *Tetraodon nigroviridis*.

Figure S4. Phylogenetic analysis of the *O. melastigma* Abcc subfamily, compared with those of other species, using the Bayesian method. Numbers at branch nodes represent the confidence level of posterior probability. Dr, *Danio rerio*; Hs, *Homo sapiens*; Ip, *Ictalurus punctatus*; Mm, *Mus musculus*; Ol, *Oryzias latipes*; Om, *Oryzias melastigma*; On, *Oreochromis niloticus*; Tn, *Tetraodon nigroviridis*.

Figure S5. Phylogenetic analysis of the *O. melastigma* Abcd subfamily, compared with those of other species, using the Bayesian method. In the case of *Danio rerio abcd2-2* and *Ictalurus punctatus abcd2*, the sequences were excluded as they disrupt overall distance values in the phylogenetic branches due to the partial cDNA sequences registered in GenBank. Numbers at branch nodes represent the confidence level of posterior probability. Dr, *Danio rerio*; Hs, *Homo sapiens*; Ip, *Ictalurus punctatus*; Mm,

Mus musculus; Ol, *Oryzias latipes*; Om, *Oryzias melastigma*; On, *Oreochromis niloticus*; Tn, *Tetraodon nigroviridis*.

Figure S6. Phylogenetic analysis of the *O. melastigma* Abce and Abcf subfamilies, compared with those of other species, using the Bayesian method. Numbers at branch nodes represent the confidence level of posterior probability. Dr, *Danio rerio*; Hs, *Homo sapiens*; Ip, *Ictalurus punctatus*; Mm, *Mus musculus*; Ol, *Oryzias latipes*; Om, *Oryzias melastigma*; On, *Oreochromis niloticus*; Tn, *Tetraodon nigroviridis*.

Figure S7. Phylogenetic analysis of the *O. melastigma* Abcg and Abch subfamilies, compared with those of other species, using the Bayesian method. Numbers at branch nodes represent the confidence level of posterior probability. Dr, *Danio rerio*; Hs, *Homo sapiens*; Ip, *Ictalurus punctatus*; Mm, *Mus musculus*; Ol, *Oryzias latipes*; Om, *Oryzias melastigma*; On, *Oreochromis niloticus*; Tn: *Tetraodon nigroviridis*.

Figure S8. Amino acid similarity of *Oryzias melastigma* and *Ictalurus punctatus abcc5* genes, which are separated into distinctive clades. Abbreviations: Om, *Oryzias melastigma*; Ip, *Ictalurus punctatus*.

Figure S9. An updated phylogenetic analysis of 50 *O. melastigma* ABC proteins with other species including arthropod *abch* genes, and three *abch* genes of invertebrates; Pacific oyster *abch* gene (*Crassostrea gigas*; EKC37771), acorn worm (*Saccoglossus kowalevskii*; XP_006817503), and sea urchin (*Strongylocentrotus purpuratus*; XP_003731550; also registered in the Sea urchin genome database (http://www.echinobase.org); ID: SPU_026438). In addition, three putative *ABCH/Abch* members were incorporated to analyze their phylogenetic locations (highlighted with yellow color; sea urchin ABC transporter H family member 2-like (*S. purpuratus*; LOC100889169), the dolphin ABC transporter H family member 2-like (*Lipotes vexillifer*; LOC103069646), and the Tibetan antelope ABC transporter H family member 2-like (*Pantholops hodgsonii*; LOC102331264). Species abbreviations: Cg, *Crassostrea gigas*; Dm, *Drosophila melanogaster*; Dp, *Daphnia pulex*; Dr, *Danio rerio*; Hs, *Homo sapiens*; Ip, *Ictalurus punctatus*; Mm, *Mus musculus*; Ol, *Oryzias latipes*; Om, *Oryzias melastigma*; On, *Oreochromis niloticus*; Sk, *Saccoglossus*

kowalevskii; Sp, *Strongylocentrotus purpuratus*; Tb, *Tribolium castaneum*; Tj, *Tigriopus japoncus*; Tn, *Tetraodon nigroviridis*.

Figure S10. Enlarged phylogenetic clade of the Abch subfamily from **Fig. S9**. Species abbreviations: Cg, *Crassostrea gigas*; Dm, *Drosophila melanogaster*; Dp, *Daphnia pulex*; Dr, *Danio rerio*; Om, *Oryzias melastigma*; Sk, *Saccoglossus kowalevskii*; Sp, *Strongylocentrotus purpuratus*; Tb, *Tribolium castaneum*; Tj, *Tigriopus japoncus*; Tn, *Tetraodon nigroviridis*.

Figure S11. Enlarged phylogenetic clade of the Abcf and Abcg subfamilies from **Fig. S9**. Species abbreviations: Dr, *Danio rerio*; Hs, *Homo sapiens*; Ip, *Ictalurus punctatus*; Mm, *Mus musculus*; Ol, *Oryzias latipes*; Om, *Oryzias melastigma*; On, *Oreochromis niloticus*; Tn, *Tetraodon nigroviridis*.

Fig. S1

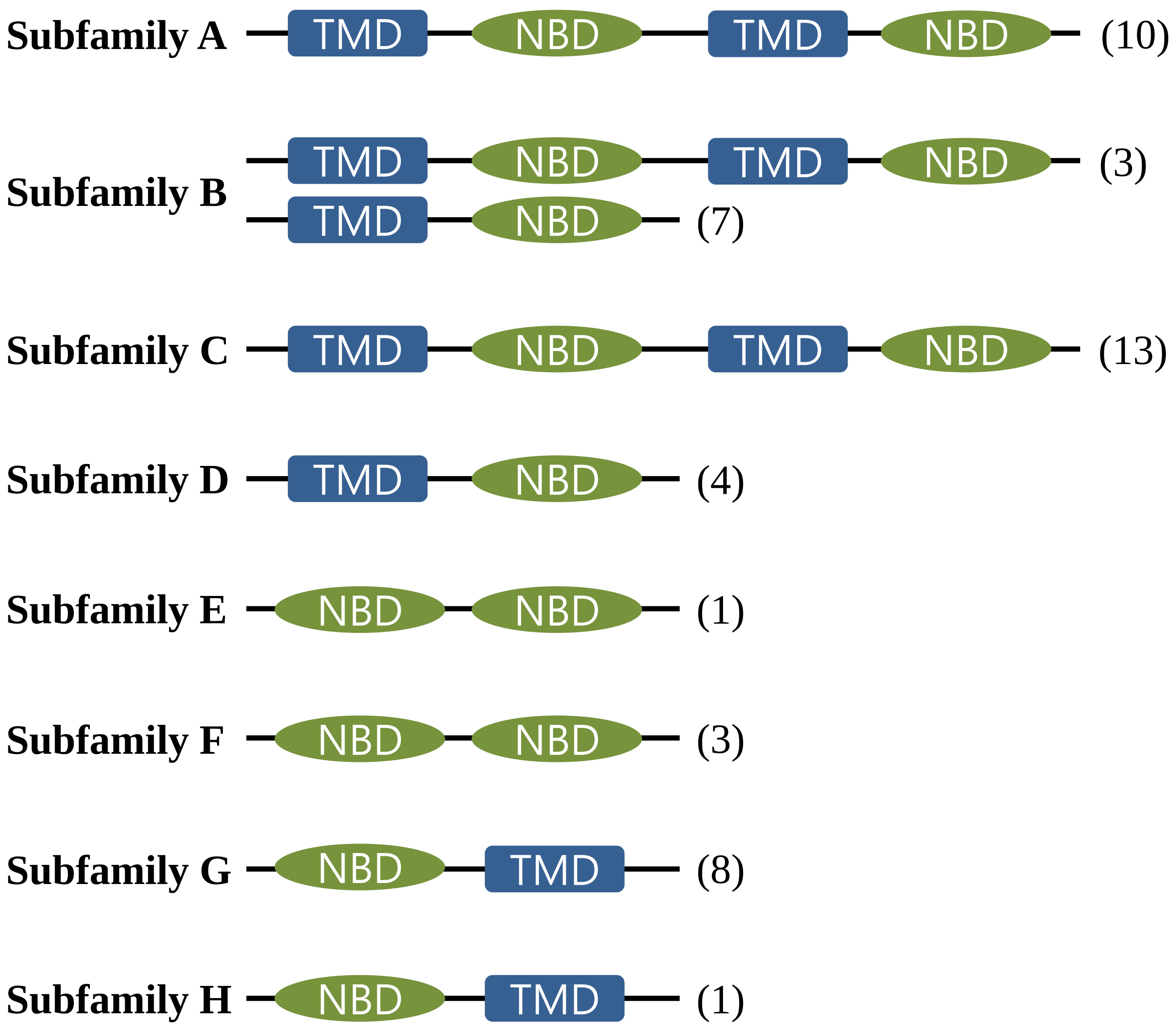
Subfamily A
TMD
NBD
TMD
NBD
(10)
Subfamily B
TMD
NBD
TMD
NBD
(3)
TMD
NBD
(7)
Subfamily C
TMD
NBD
TMD
NBD
(13)
Subfamily D
TMD
NBD
(4)
Subfamily E
NBD
NBD
(1)
Subfamily F
NBD
NBD
(3)
Subfamily G
NBD
TMD
(8)
Subfamily H
NBD
TMD
(1)

Subfamily A

Fig. S2

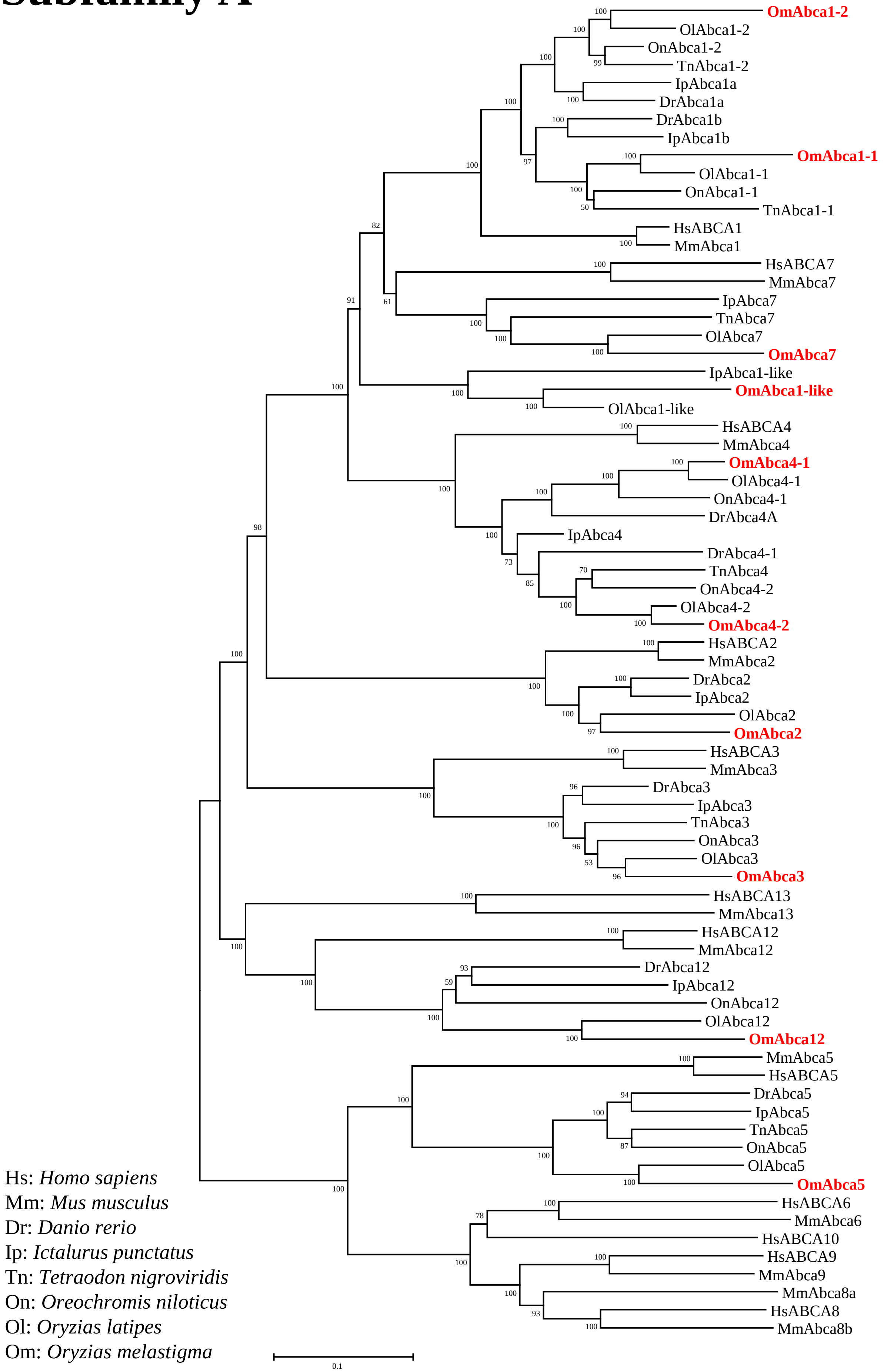

Subfamily B

Fig. S3

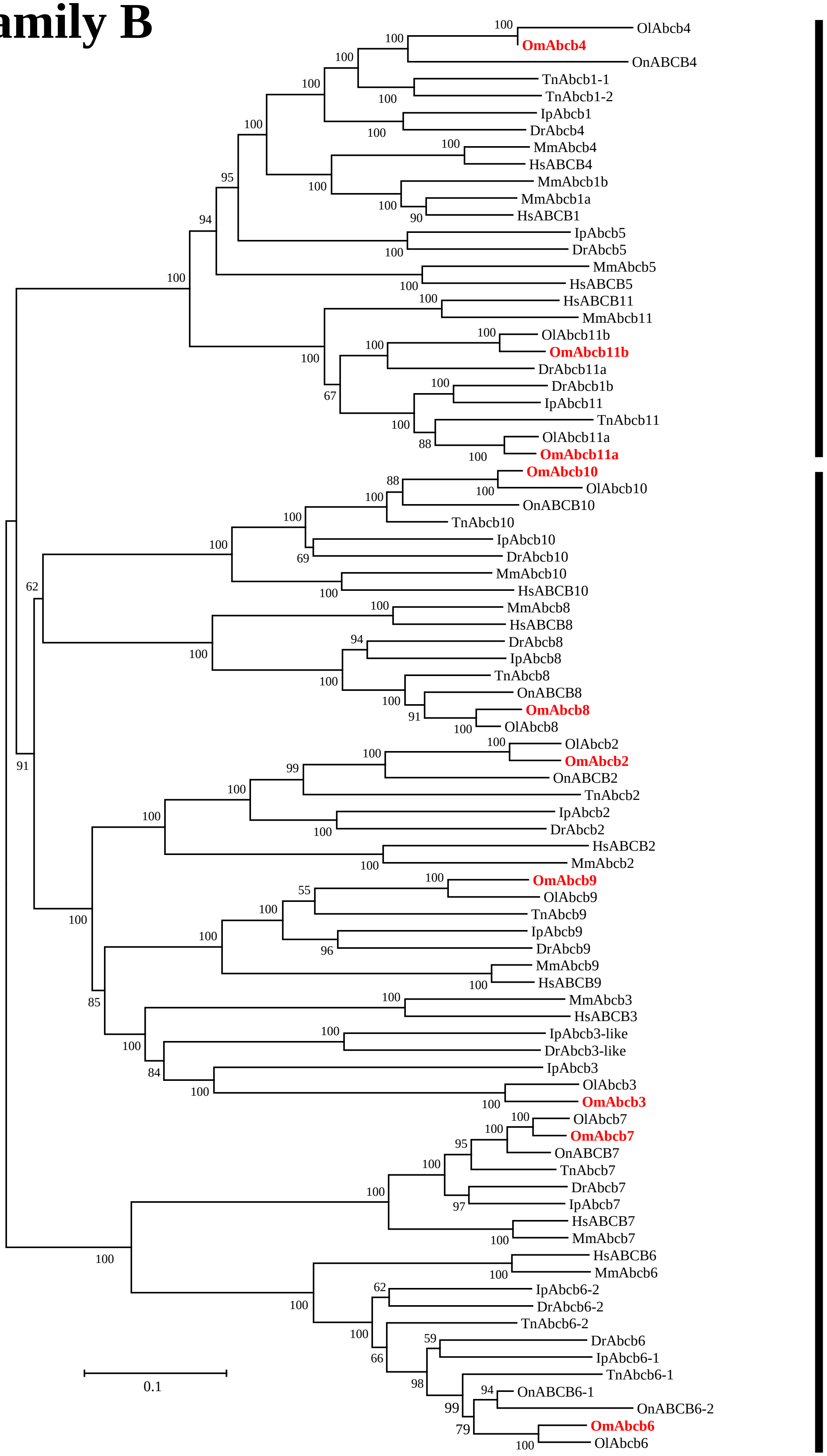

Subfamily C

Fig. S4

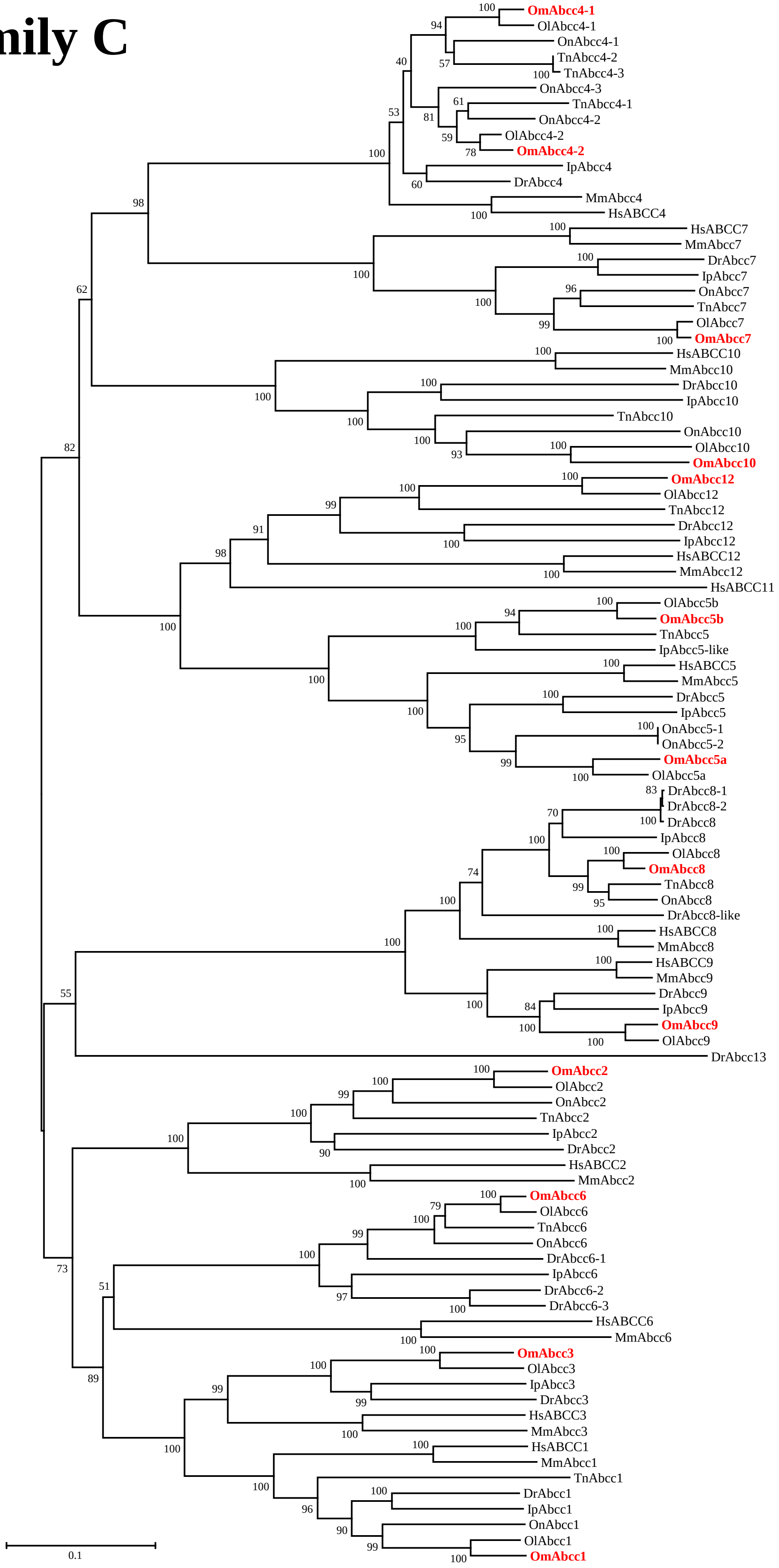

Subfamily D

Fig. S5

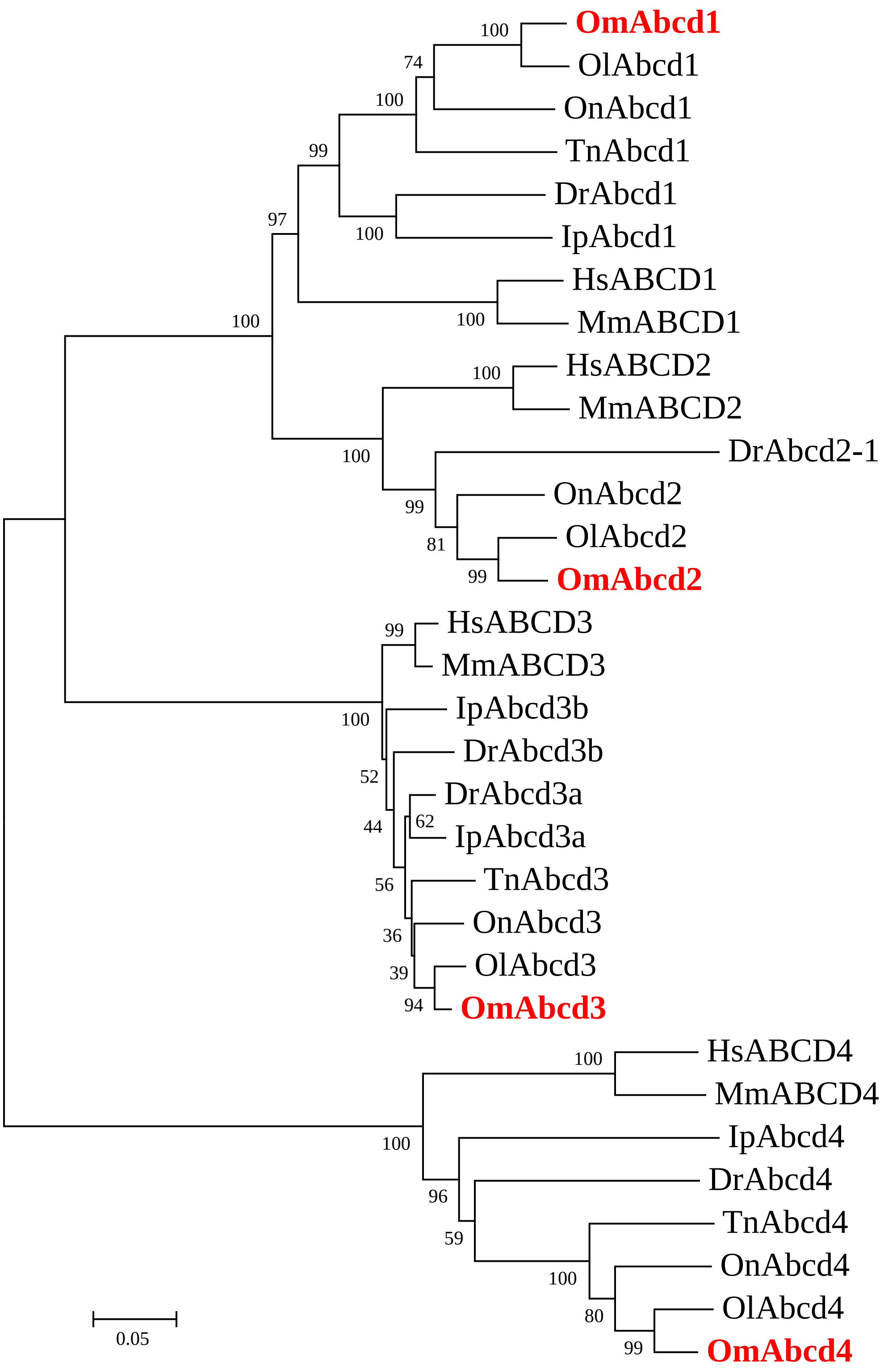

Subfamily E and F

Fig. S6

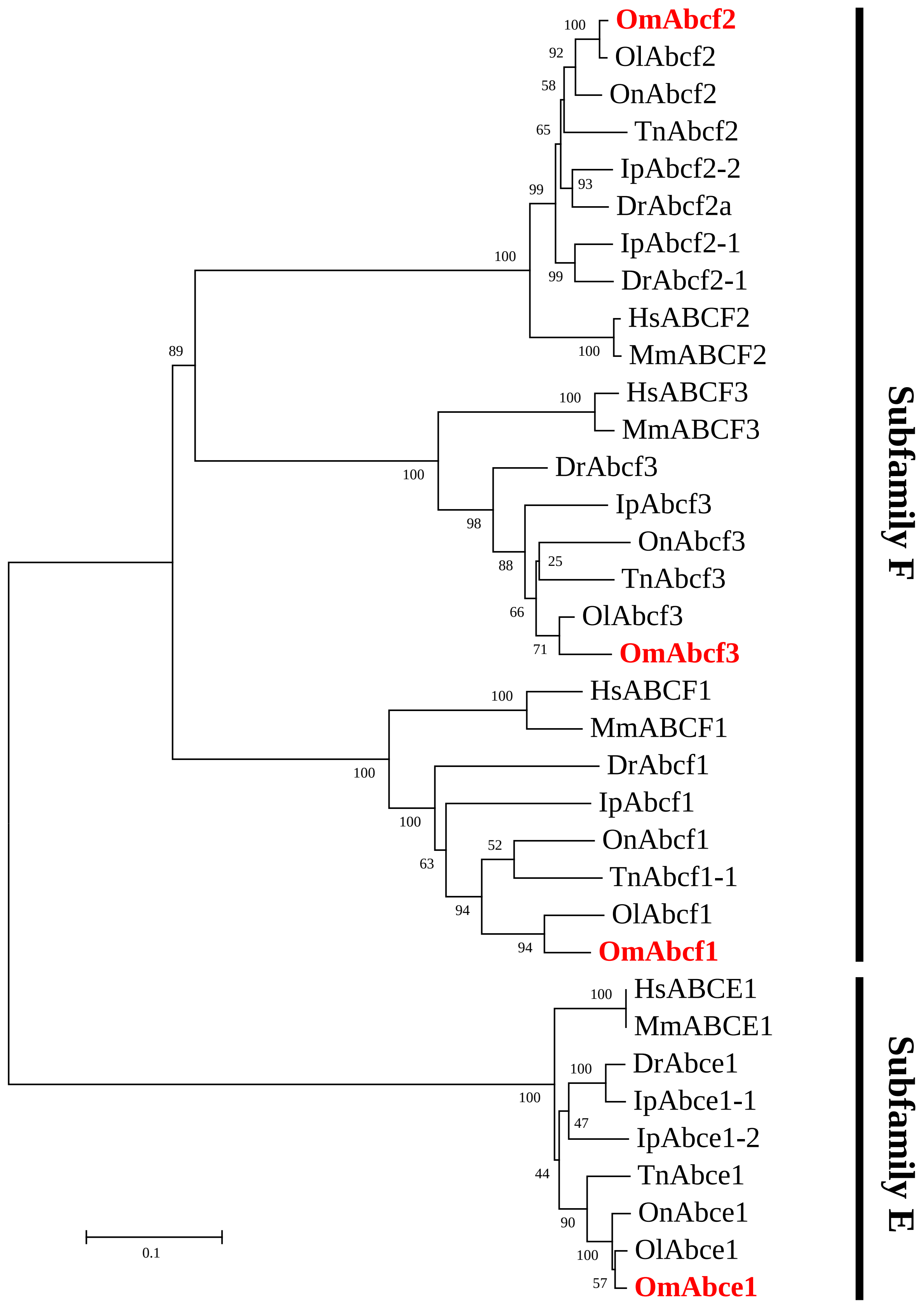

Subfamily G and H

Fig. S7

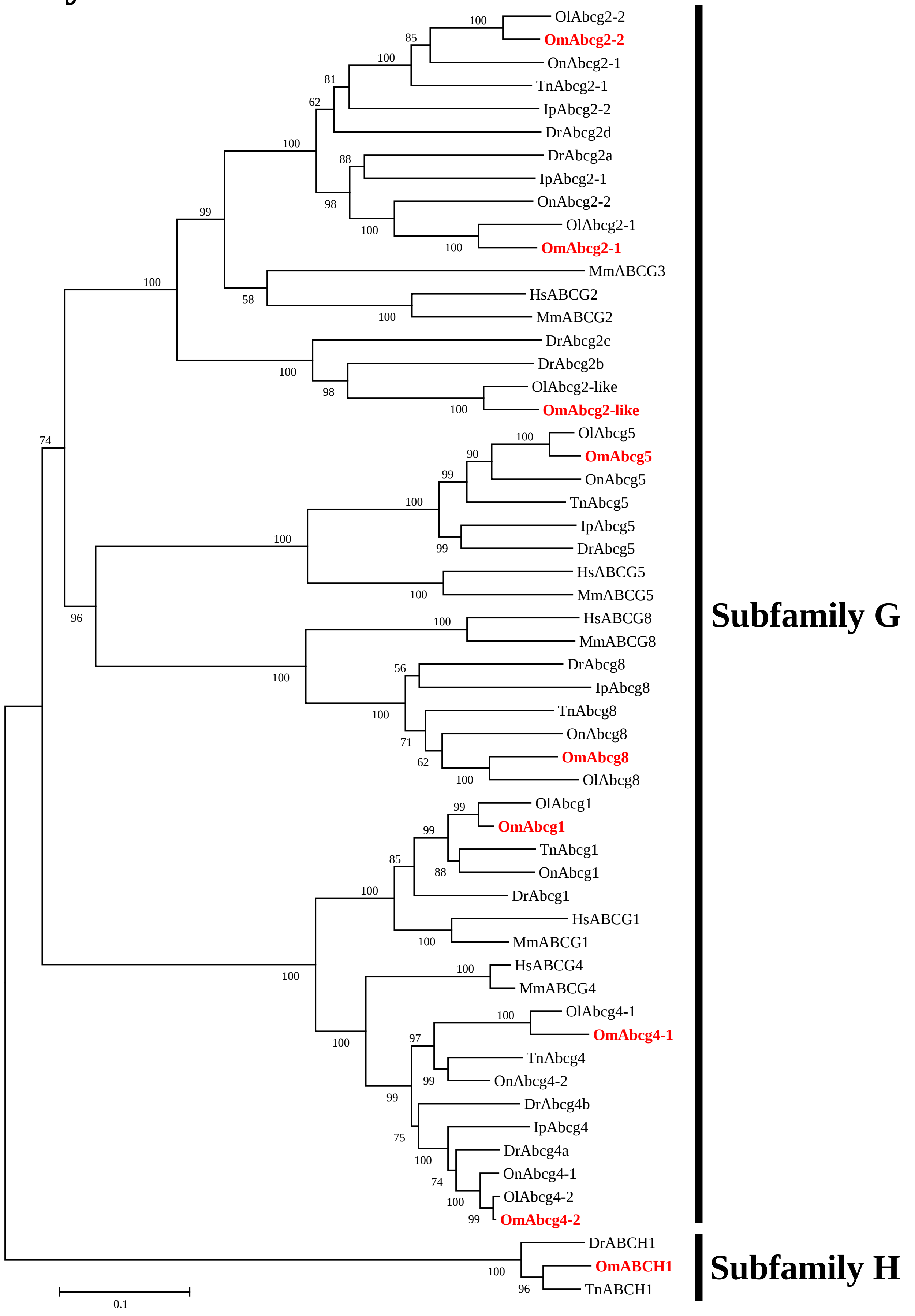

Fig. S8

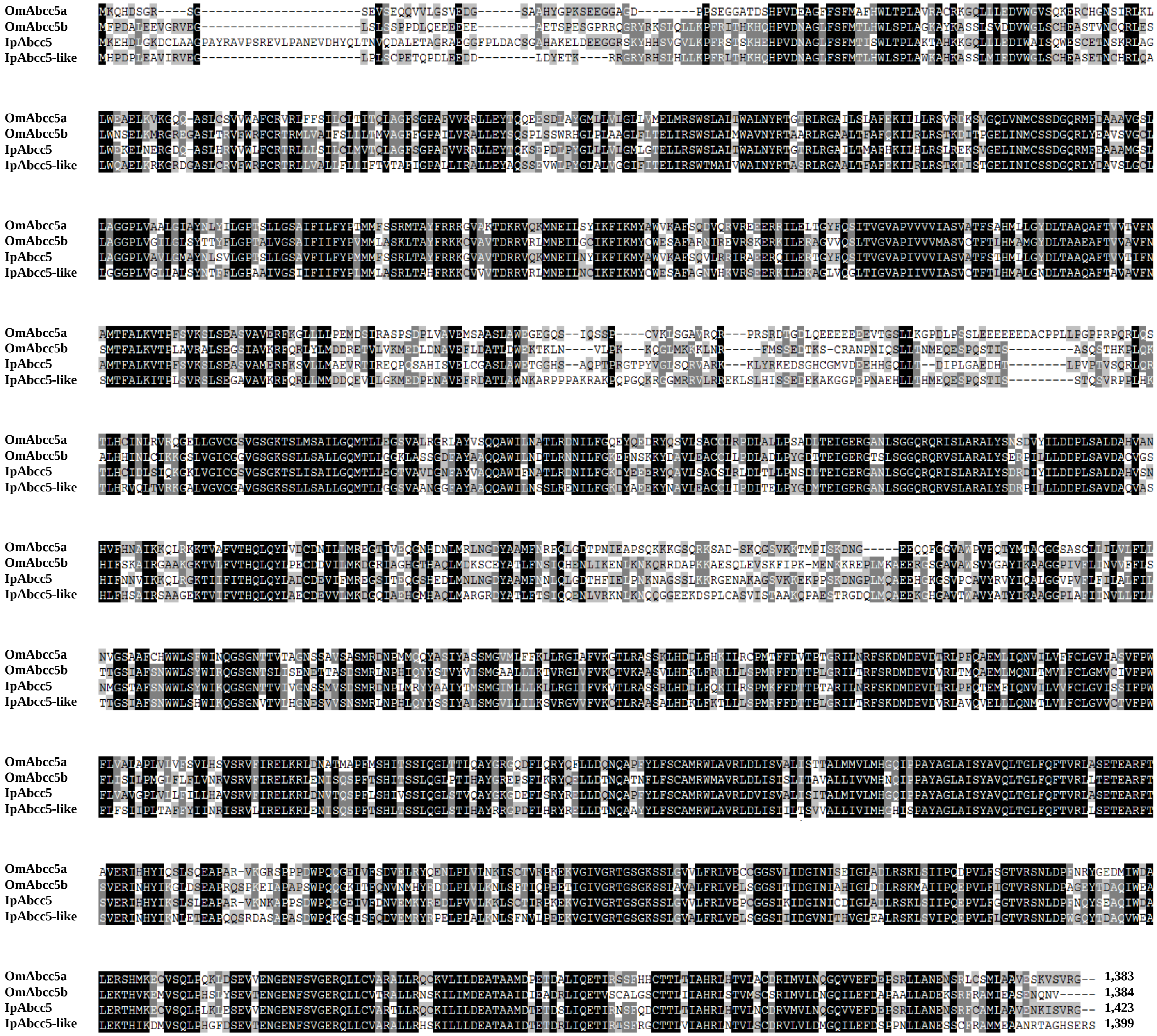
OmAbcc5a
OmAbcc5b
IpAbcc5
IpAbcc5-like
1,383
1,384
1,423
1,399

Fig. S9

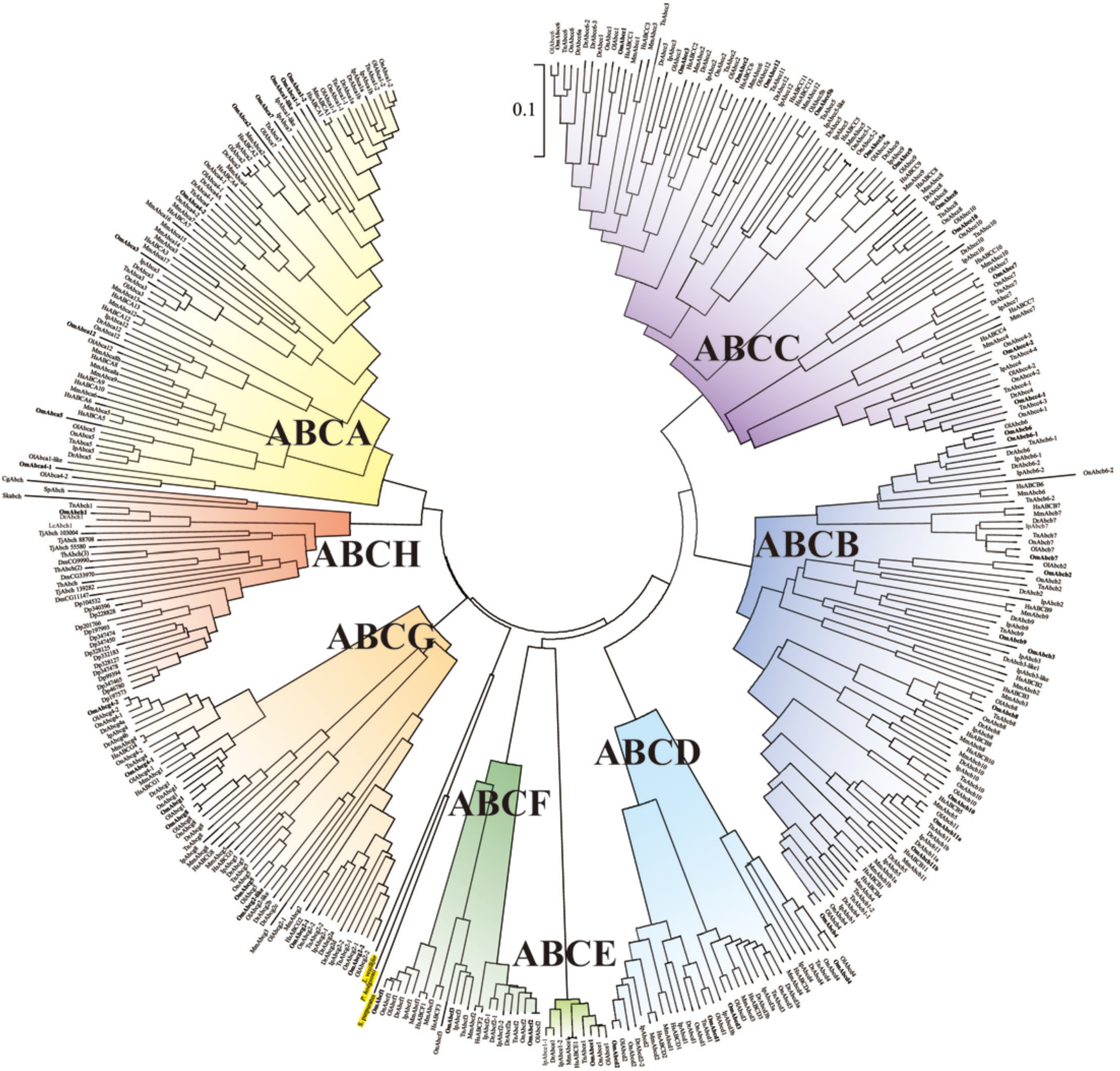

Fig. S10

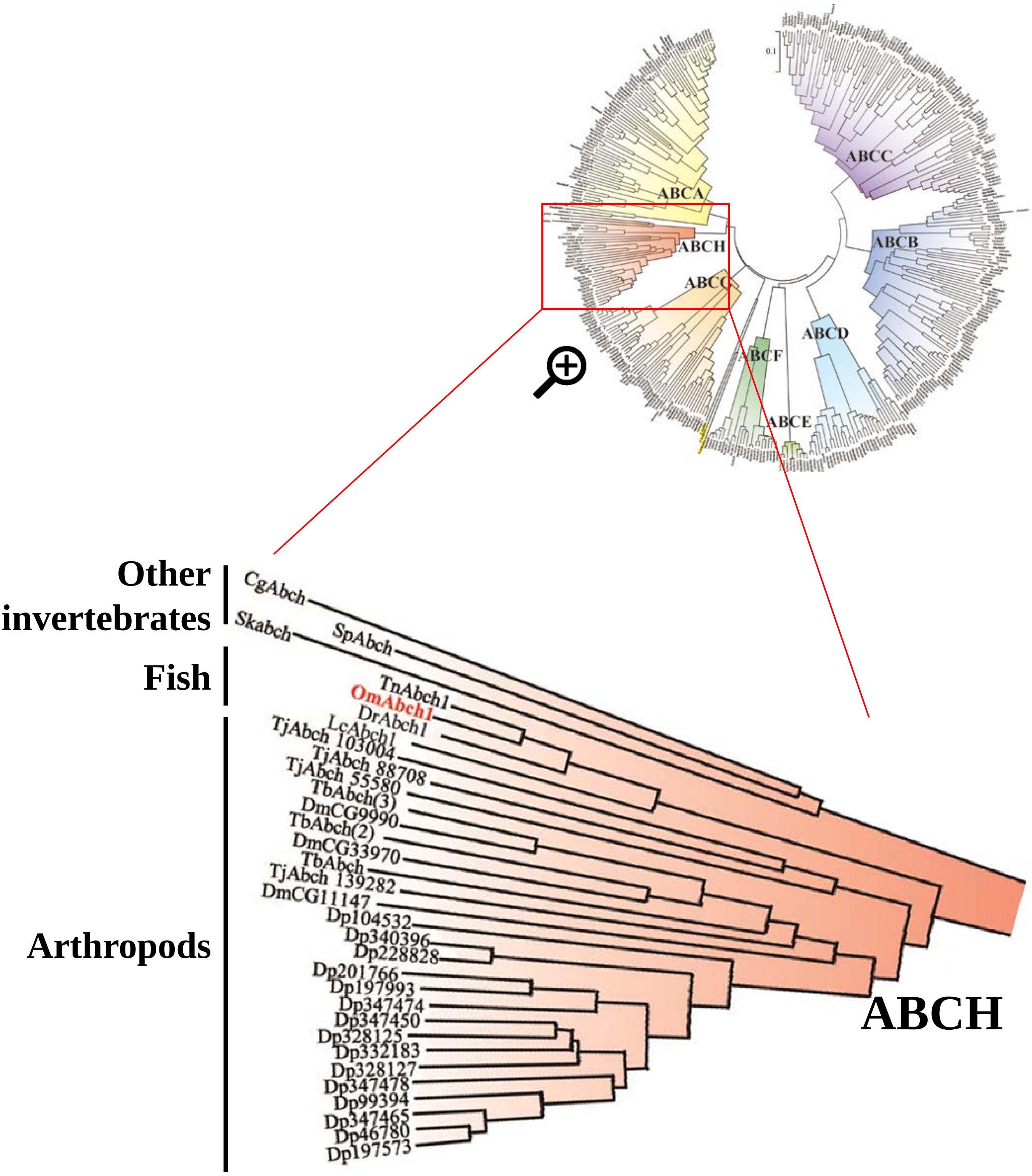
ABCC
ABCA
ABCH
ABCG
ABCB
ABCD
ABCF
ABCE
Other invertebrates
Fish
Arthropods
CgAbch
Skabch
SpAbch
TnAbch1
OmAbch1
DrAbch1
LcAbch1
TjAbch 103004
TjAbch 88708
TjAbch 55580
TbAbch(3)
DmCG9990
TbAbch(2)
DmCG33970
TbAbch
TjAbch 139282
DmCG11147
Dp104532
Dp340396
Dp228828
Dp201766
Dp197993
Dp347474
Dp347450
Dp328125
Dp332183
Dp328127
Dp347478
Dp99394
Dp347465
Dp46780
Dp197573
ABCH

Fig. S11

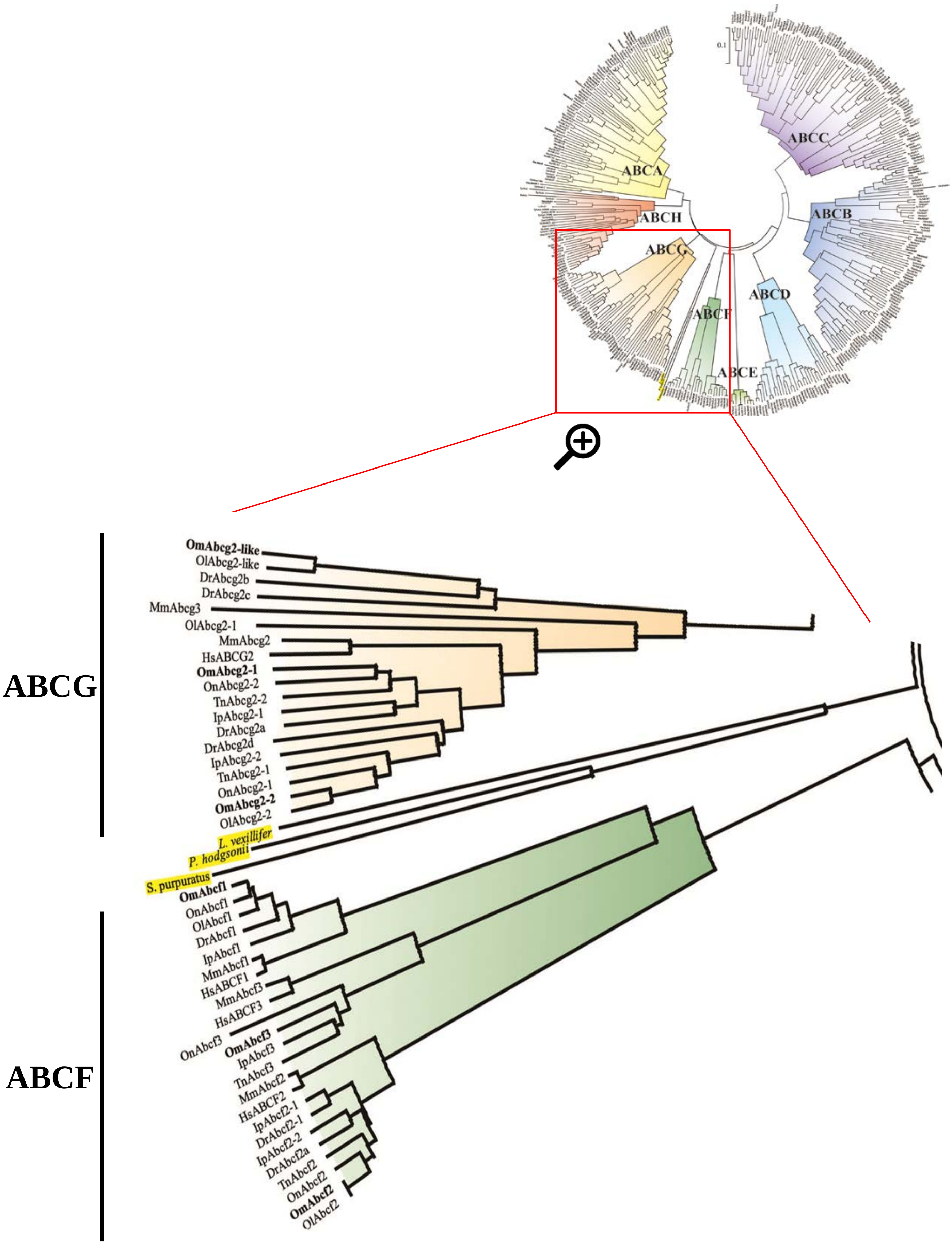
ABCA
ABCC
ABCH
ABCB
ABCG
ABCD
ABCF
ABCE
0.1
OmAbcg2-like
OlAbcg2-like
DrAbcg2b
DrAbcg2c
MmAbcg3
OlAbcg2-1
MmAbcg2
HsABCG2
OmAbcg2-1
OnAbcg2-2
TnAbcg2-2
IpAbcg2-1
DrAbcg2a
DrAbcg2d
IpAbcg2-2
TnAbcg2-1
OnAbcg2-1
OmAbcg2-2
OlAbcg2-2
L. vexillifer
P. hodgsonii
S. purpuratus
OmAbcf1
OnAbcf1
OlAbcf1
DrAbcf1
IpAbcf1
MmAbcf1
HsABCF1
MmAbcf3
HsABCF3
OnAbcf3
OmAbcf3
IpAbcf3
TnAbcf3
MmAbcf2
HsABCF2
IpAbcf2-1
DrAbcf2-1
IpAbcf2-2
DrAbcf2a
TnAbcf2
OnAbcf2
OmAbcf2
OlAbcf2
